# Association between genetic risk and adherence to healthy lifestyle for developing age-related hearing loss

**DOI:** 10.1186/s12916-024-03364-5

**Published:** 2024-03-26

**Authors:** Sang-Hyuk Jung, Young Chan Lee, Manu Shivakumar, Jaeyoung Kim, Jae-Seung Yun, Woong-Yang Park, Hong-Hee Won, Penn Medicine Biobank, Dokyoon Kim

**Affiliations:** 1 Department of Biostatistics, Epidemiology and Informatics, Perelman School of Medicine, University of Pennsylvania, Philadelphia, PA USA; 2 Department of Otolaryngology-Head and Neck Surgery, School of Medicine, Kyung Hee University, Kyung Hee University Hospital at Gangdong, Seoul, Republic of Korea; 3 Samsung Advanced Institute for Health Sciences and Technology (SAIHST), Sungkyunkwan University, Samsung Medical Center, Seoul, Republic of Korea; 4 Division of Endocrinology and Metabolism, Department of Internal Medicine, St. Vincent’s Hospital, College of Medicine, The Catholic University of Korea, Seoul, Republic of Korea; 5 Samsung Genome Institute, Samsung Medical Center, Sungkyunkwan University School of Medicine, Seoul, Republic of Korea; 6 Institute for Translational Medicine and Therapeutics, Perelman School of Medicine, University of Pennsylvania, Philadelphia, PA USA; 7 Institute for Biomedical Informatics, University of Pennsylvania, Philadelphia, USA

**Keywords:** Age-related hearing loss, Genetic risk, Polygenic risk score, Lifestyle, Environmental factor

## Abstract

**Background:**

Previous studies have shown that lifestyle/environmental factors could accelerate the development of age-related hearing loss (ARHL). However, there has not yet been a study investigating the joint association among genetics, lifestyle/environmental factors, and adherence to healthy lifestyle for risk of ARHL. We aimed to assess the association between ARHL genetic variants, lifestyle/environmental factors, and adherence to healthy lifestyle as pertains to risk of ARHL.

**Methods:**

This case–control study included 376,464 European individuals aged 40 to 69 years, enrolled between 2006 and 2010 in the UK Biobank (UKBB). As a replication set, we also included a total of 26,523 individuals considered of European ancestry and 9834 individuals considered of African-American ancestry through the Penn Medicine Biobank (PMBB). The polygenic risk score (PRS) for ARHL was derived from a sensorineural hearing loss genome-wide association study from the FinnGen Consortium and categorized as low, intermediate, high, and very high. We selected lifestyle/environmental factors that have been previously studied in association with hearing loss. A composite healthy lifestyle score was determined using seven selected lifestyle behaviors and one environmental factor.

**Results:**

Of the 376,464 participants, 87,066 (23.1%) cases belonged to the ARHL group, and 289,398 (76.9%) individuals comprised the control group in the UKBB. A very high PRS for ARHL had a 49% higher risk of ARHL than those with low PRS (adjusted OR, 1.49; 95% CI, 1.36–1.62; *P* < .001), which was replicated in the PMBB cohort. A very poor lifestyle was also associated with risk of ARHL (adjusted OR, 3.03; 95% CI, 2.75–3.35; *P* < .001). These risk factors showed joint effects with the risk of ARHL. Conversely, adherence to healthy lifestyle in relation to hearing mostly attenuated the risk of ARHL even in individuals with very high PRS (adjusted OR, 0.21; 95% CI, 0.09–0.52; *P* < .001).

**Conclusions:**

Our findings of this study demonstrated a significant joint association between genetic and lifestyle factors regarding ARHL. In addition, our analysis suggested that lifestyle adherence in individuals with high genetic risk could reduce the risk of ARHL.

**Supplementary Information:**

The online version contains supplementary material available at 10.1186/s12916-024-03364-5.

## Background

Age-related hearing loss (ARHL), also known as presbycusis, is a disease of complex etiology resulting from the cumulative effects of aging on auditory function, although the underlying mechanisms of ARHL remain incompletely elucidated. It is characterized by hearing difficulty in the high-frequency sound range and has a bilateral, symmetrical, progressive pattern [1]. According to the World Health Organization, the prevalence of moderate to severe hearing loss (HL) increases exponentially with age worldwide [2]. ARHL can develop into a common social and health problem, and untreated HL can lead to social isolation, depression, and loss of self-esteem [3, 4]. Therefore, ARHL can have significant adverse effects on the quality of life in older adults and can be perceived as a serious disability for the elderly even when mild [5, 6]. ARHL may be caused by aging of the cochlea, specifically the development of synaptopathy between sensory hair cells and cochlea nerve fibers [7]. Additionally, synaptopathy can be induced by pre-existing ear conditions, chronic disease, noise exposure, ototoxic drugs, and lifestyle, along with genetic factors [8, 9].

In 2009, the first genome-wide association study (GWAS) on ARHL was reported with 1692 participants, and several further GWAS concerning hearing status have since been published [10–14]. However, while many single-nucleotide polymorphisms (SNPs) have been linked to ARHL, their impacts are limited. Recently, polygenic risk score (PRS) has been widely used to predict complex traits or diseases in humans by summarizing the effects of genetic variants across the genome [15]. Cherny et al. demonstrated that a PRS calculated from UK Biobank (UKBB) GWAS results could predict HL status in the TwinsUK cohort as defined from questionnaire and hearing test results [16]. Previous studies have reported associations of HL with several modifiable environmental factors including noise exposure, smoking, alcohol, and comorbidity [17–19]. In addition, it has been shown that healthy lifestyle behaviors could attenuate the development of HL [17]. This suggests that gene-environment interactions and epigenetic changes may play important roles in regulating the genes that specifically affect aging-related traits [20].

Despite these reports, there is to our knowledge no study that has investigated the joint association between genetics and lifestyle behavior in relation to risk of ARHL. Therefore, we constructed a PRS for ARHL and assessed its performance in two independent datasets. We further investigated the association between lifestyle behavior and genetic risk for ARHL in UKBB participants. Finally, we demonstrated that healthy lifestyle behavior could reduce the development of the disease in individuals having a high genetic predisposition.

## Methods

### Study population

The UKBB is a large prospective observational cohort study that has recruited > 500,000 adults across 22 centers located throughout the UK. The full protocol of the UKBB study is publicly available, and the study design and measurement methods have been described elsewhere [21]. Participants aged 40–69 years were enrolled between 2006 and 2010 and were followed up with for subsequent health events. We excluded individuals with any single International Classification of Diseases (ICD)-10 code for conductive HL or a congenital disorder that causes impairment of hearing (H90.0, H90.1, H90.2, H91.3, Q16.1, Q16.3, Q16.4, Q16.5, or Q16.9) at the baseline period (*n* = 626). All ICD-9 and ICD-10 diagnosis codes, and laboratory measurements up to July 2020 were extracted from the electronic health records (EHRs).

The Penn Medicine Biobank (PMBB) is a large academic medical biobank in which participants are agnostically recruited from the outpatient setting and consented for access to their EHR data and permission to generate genomic and biomarker data [22]. The study flowchart is illustrated in Additional file 1: Fig. S1.

### Definition of ARHL

For UKBB participants, we defined AHRL according to self-report questionnaires, which have previously been found useful for large-scale study of HL [16]. If a participant answered, ‘Yes’ to ‘Do you use a hearing aid most of the time?’ or ‘Yes’ to both ‘Do you have any difficulty with your hearing?’ and ‘Do you find it difficult to follow a conversation if there is background noise (such as TV, radio, children playing)?’, they were classified as an ARHL case. Participants who answered ‘No’ to all these questions were classified as controls. Individuals who selected the answer ‘I am completely deaf’ or declined to answer were excluded. For the PMBB, we classified ARHL cases using ICD-9 or ICD-10 codes in the EHR system. The detailed definition criteria of ARHL in each cohort are described in Additional file 1: Method S2. [11, 23–25].

### Definitions of variables

#### Covariate definition

We included several covariates, including demographics, biomarkers, body compositions, sociodemographic characteristics, and major chronic comorbidities, as potential confounding factors in the ARHL association analyses. A detailed description of the considered covariates can be found in Additional file 1: Methods S3 and S4 [21, 26].

#### Lifestyle and environmental factors

During the enrollment process in the UKBB, participants provided information on their sociodemographic characteristics, health/medical history, and lifestyle/environmental factors through a self-administered touchscreen questionnaire and in-person baseline interviews. We selected lifestyle/environmental factors that have been previously studied in association with HL [17, 18, 23, 24] (Additional file 1: Method S5).

#### Healthy lifestyle score

We developed a composite healthy lifestyle score (HLS), which provides a comprehensive measure of lifestyle-related risk factors for ARHL, based on seven selected lifestyle behaviors and one environmental factor: Listening to music (loud music exposure frequency, Field ID: 4836), Computer games (Field ID: 2237), Obesity (body mass index [BMI] at baseline), Smoking history (Never/Ever, Field ID: 20116), Alcohol history (Never/Ever, Field ID:20117), Use of ototoxic drugs (aspirin and/or ibuprofen consumption, Field ID: 6154), Sleep (Sleeplessness/insomnia, Field ID: 1200), and Noisy workplace (Field ID: 4825). We excluded participants with missing variables required for constructing the composite HLS, a total of 85,588 participants eligible for the joint analysis with composite HLS (Additional file 1: Fig. S2 and Method S6). To generate the HLS, each variable was assigned a score of 0 or 1, with 1 representing a healthy behavior. Participant lifestyles as reflected by the HLS were categorized into four groups: very poor (0–2 healthy behaviors), poor (3–4 healthy behaviors), intermediate (5–6 healthy behaviors), and ideal (≥ 7 healthy behaviors). Detailed information on the HLS is given in Additional file 1: Method S7.

#### AHA lifestyle and MetS health scores

To compare the proposed HLS with the previously used lifestyle score, we generated an American Heart Association (AHA) lifestyle score and metabolic syndrome (MetS) health score based on the International Diabetes Federation consensus report [29–32]. According to AHA, five factors are primarily considered to define lifestyle behaviors: current smoking, alcohol consumption, obesity, physical activity, and eating habits. Collectively, lifestyle behaviors are categorized into three groups: poor (0–1 healthy lifestyle factor), intermediate (2 healthy lifestyle factors), and ideal (≥ 3 healthy lifestyle factors). MetS health score was categorized into three groups: ideal (0–1 MetS factor), intermediate (2–3 MetS factors), and poor (≥ 4 MetS factors). Detailed descriptions and definitions of the variables considered in scores can be found in Additional file 1: Method S8.

### Genotype data quality control and imputation

Genotyping and quality control (QC) procedures and imputation followed standard practices and were performed per cohort-genotyping platform pair. We have filtered out related individuals (with second-degree or closer relatives) in both biobanks.

#### UK Biobank

UKBB samples (version 3; March 2018) were genotyped for > 800,000 SNPs using either the Affymetrix UK BiLEVE Axiom array or the Affymetrix UKBB Axiom array. Imputation was carried out centrally by UK Biobank researchers using the merged 1000 Genomes Project panel and UK 10K panel; SHAPEIT3 was used for phasing and IMPUTE2 was used for imputation (GRCh37/hg19) [33, 34]. After QC and imputation, 376,464 European (White-British) individuals were determined eligible for the validation genetic analyses. Further details are described in Additional file 1: Method S9 [33–39].

#### Penn Medicine Biobank

The PMBB consists of 43,623 samples that have been genotyped with the GSA genotyping array. We performed genotype imputation for the PMBB dataset using Eagle2 and Minimac4 software on TOPMed Imputation Server [35–37]. After QC and imputation, a total of 26,523 individuals considered of European (non-Hispanic White) ancestry and 9834 individuals considered of African American (non-Hispanic Black) ancestry were determined eligible for the genetic replication analyses. Further details are described in Additional file 1: Method S9 [33–39].

#### Polygenic risk score

The HL PRS was generated based on the large-scale sensorineural HL GWAS summary statistics (28,310 cases and 302,750 controls) from the FinnGen Consortium (Data Freeze R8v4) using the Bayesian polygenic prediction method PRS-CS [40, 41]. Individual PRSs were computed from beta coefficients as the weighted sum of the risk alleles by applying PLINK version 1.90 with the –score command [42]. Additionally, we generated PRSs using several alternative methods, including LDpred2, lassosum, and PRSice-2, and compared their performance. Details of the PRS analysis are described in Additional file 1: Method S10 [40–47].

## Statistical analysis

Demographic and clinical characteristics are presented as mean ± standard deviation (SD) or as number (percentage). Continuous variables were compared by Student’s *t* test or the Mann–Whitney *U* test as appropriate. Categorical variables were compared by the chi-square test or Fisher’s exact test as appropriate.

We used a multivariate logistic regression model to evaluate the association of the HL PRS and lifestyle/environmental factors with ARHL. In the primary analysis, we calculated odds ratios (ORs) and 95% confidence intervals (CIs) after adjusting for age, sex, the first ten principal components (PCs) of ancestry, and genotyping array type. The PRS was categorized as follows: low (< 20%), intermediate (20–80%), high (80–99%), and very high (> 99%) risk groups to quantitatively assess the ARHL risk. The low (< 20%) risk group bin was used as the reference to estimate relative ORs across PRS risk increases. In sensitivity analyses, regression models were additionally adjusted for baseline demographics and major chronic comorbidities. Subsequently, we conducted joint association and multiplicative interaction analyses to investigate the interplay between genetic and lifestyle/environmental factors.

We further performed a Cox proportional hazards regression analysis in PMBB participants with age at ARHL onset and age at the last clinical visit as the time variables and ARHL diagnosis as the status; with this, we calculated hazard ratios (HRs) and 95% CIs. Kaplan–Meier curves were then conducted to check if survival differed significantly between genetic risk groups.

All statistical tests were two-sided, and *P* < 0.05 was considered statistically significant. All statistical analyses were conducted using the R Statistical Software (version 4.1.0; R Foundation for Statistical Computing, Vienna, Austria) and PLINK version 1.90 [44]. Details of the statistical analyses are described in Additional file 1: Method S11.

## Results

### Population characteristics

In total, 376,464 participants who did not have conductive or congenital HL history were included in this study. The mean age of participants was 57.5 years (SD, 7.9 years), and 46.3% were men. Of included participants, 87,066 (23.1%) were cases (the ARHL group) and 289,398 (76.9%) were controls. A comparison of participant characteristics in each group is presented in Table 1. Baseline demographics and clinical characteristics stratified by PRS group, as well as according to composite HLS analysis inclusion criteria are given in Additional file 1: Tables S1 and Additional file 1: Table S2, respectively.

**Table 1:** Characteristics of participants in the UK Biobank

	**Total** **(*n* = 376,464)**	**Control** **(*n* = 289,398)**	**ARHL case** **(*n* = 87,066)**	*P*-value^*^
Age, mean (SD), years	57.5 ± 7.9	56.8 ± 8.0	59.6 ± 7.3	< .001
Sex, No. (%)				< .001
Male	174,205 (46.3%)	124,974 (43.2%)	49,231 (56.5%)	
Female	202,259 (53.7%)	164,424 (56.8%)	37,835 (43.5%)	
Education years, mean (SD), years	13.8 ± 5.1	13.8 ± 5.1	13.7 ± 5.2	< .001
Number in household, mean (SD)	2.4 ± 1.2	2.4 ± 1.3	2.3 ± 1.2	< .001
Townsend deprivation index, mean (SD)	− 1.6 ± 2.9	− 1.6 ± 2.9	− 1.5 ± 3.0	< .001
Average total household income before tax				< .001
Less than £18,000	71,597 (22.1%)	52,299 (21.0%)	19,298 (25.7%)	
18,000 to 30,999£	83,698 (25.8%)	63,034 (25.3%)	20,664 (27.5%)	
31,000 to 51,999£	85,554 (26.4%)	66,666 (26.8%)	18,888 (25.2%)	
52,000 to 100,000£	66,229 (20.4%)	53,021 (21.3%)	13,208 (17.6%)	
Greater than 100,000£	16,994 (5.2%)	13,983 (5.6%)	3011 (4.0%)	
***Body composition***				
Body mass index, mean (SD), kg/m^2^	27.4 ± 4.8	27.3 ± 4.8	27.8 ± 4.7	< .001
Height, mean (SD), cm	168.8 ± 9.3	168.5 ± 9.2	169.6 ± 9.3	< .001
Weight, mean (SD), kg	78.3 ± 15.9	77.8 ± 15.8	80.3 ± 15.9	< .001
Waist circumference, mean (SD), cm	90.4 ± 13.5	89.7 ± 13.4	92.7 ± 13.4	< .001
Systolic blood pressure, mean (SD), mmHg	140.3 ± 19.7	139.9 ± 19.7	141.5 ± 19.4	< .001
Diastolic blood pressure, mean (SD), mmHg	82.3 ± 10.7	82.3 ± 10.7	82.4 ± 10.6	.005
***Hearing condition***				
Speech reception threshold (SRT), No. (%)				< .001
Normal (SRT < − 5.5 dB)	120,676 (79.1%)	93,660 (83.5%)	27,016 (66.9%)	
Insufficient (− 5.5 dB to − 3.5 dB)	25,559 (6.8%)	15,901 (14.2%)	9658 (23.9%)	
Poor (SRT > − 3.5 dB)	6262 (4.1%)	2577 (2.3%)	3685 (9.1%)	
Tinnitus, No. (%)				< .001
No, never	86,181 (70.8%)	70,981 (77.2%)	15,200 (51.1%)	
Yes, but not now, but have in the past	13,382 (11.0%)	9503 (10.3%)	3879 (13.0%)	
Yes, now some of the time	10,913 (9.0%)	6706 (7.3%)	4207 (14.1%)	
Yes, now a lot of the time	3114 (2.6%)	1557 (1.7%)	1557 (5.2%)	
Yes, now most or all of the time	8072 (6.6%)	3155 (3.4%)	4917 (16.5%)	
Tinnitus severity/nuisance, No. (%)				< .001
Not at all	11,412 (32.4%)	7750 (37.4%)	3662 (25.3%)	
Slightly	16,980 (48.2%)	9987 (48.1%)	6993 (48.3%)	
Moderately	5766 (16.4%)	2590 (12.5%)	3176 (21.9%)	
Severely	1075 (3.1%)	415 (2.0%)	660 (4.6%)	
***Environmental factor***				
Noisy workplace, No. (%)				< .001
No	93,779 (76.4%)	74,566 (80.4%)	19,213 (64.0%)	
Yes, for less than a year	6681 (5.4%)	4892 (5.3%)	1789 (6.0%)	
Yes, for around 1–5 years	7068 (5.8%)	4775 (5.1%)	2293 (7.6%)	
Yes, for more than 5 years	15,268 (12.4%)	8566 (9.2%)	6702 (22.3%)	
Workplace very noisy, No. (%)				< .001
Rarely/never	51,657 (54.8%)	40,018 (56.2%)	11,639 (50.6%)	
Sometimes	33,097 (35.1%)	24,852 (34.9%)	8245 (35.8%)	
Often	9469 (10.0%)	6351 (8.9%)	3118 (13.6%)	
Daytime sound level of noise pollution, mean (SD), dB	55.3 ± 4.2	55.3 ± 4.2	55.3 ± 4.2	.530
Evening sound level of noise pollution, mean (SD), dB	51.6 ± 4.2	51.6 ± 4.2	51.6 ± 4.2	.531
Night-time sound level of noise pollution, mean (SD), dB	46.5 ± 4.2	46.5 ± 4.2	46.5 ± 4.2	.530
16-h sound level of noise pollution, mean (SD), dB	54.4 ± 4.2	54.4 ± 4.2	54.4 ± 4.2	.530
24-h sound level of noise pollution, mean (SD), dB	56.0 ± 4.2	56.0 ± 4.2	56.0 ± 4.2	.530
***Lifestyle factor***				
Loud music exposure frequency, No. (%)				< .001
No	107,501 (88.0%)	82,466 (89.2%)	25,035 (84.1%)	
Yes, for less than a year	3728 (3.1%)	2719 (2.9%)	1009 (3.4%)	
Yes, for around 1–5 years	5692 (4.7%)	3874 (4.2%)	1818 (6.1%)	
Yes, for more than 5 years	5285 (4.3%)	3388 (3.7%)	1897 (6.4%)	
Plays computer games, No. (%)				< .001
Rarely/never	292,742 (77.8%)	225,755 (78.0%)	66,987 (77.0%)	
Sometimes	69,425 (18.5%)	53,254 (18.4%)	16,171 (18.6%)	
Often	14,108 (3.7%)	10,249 (3.5%)	3859 (4.4%)	
Sleeplessness/insomnia, No. (%)				< .001
Rarely/never	82,336 (21.9%)	66,033 (22.8%)	16,303 (18.7%)	
Sometimes	178,250 (47.4%)	138,822 (48.0%)	39,428 (45.3%)	
Usually	115,588 (30.7%)	84,314 (29.2%)	31,274 (35.9%)	
Alcohol drinker status, No. (%)				< .001
Never	11,452 (3.0%)	9085 (3.1%)	2367 (2.7%)	
Previous	12,697 (3.4%)	9464 (3.3%)	3233 (3.7%)	
Current	351,947 (93.6%)	270,555 (93.6%)	81,392 (93.6%)	
Smoking status, No. (%)				< .001
Never	204,200 (54.4%)	161,672 (56.1%)	42,528 (49.0%)	
Previous	133,033 (35.5%)	97,518 (33.8%)	35,515 (40.9%)	
Current	37,957 (10.1%)	29,246 (10.1%)	8711 (10.0%)	
***Laboratory result***				
Total cholesterol, mean (SD), mmol/l	220.7 ± 44.2	221.4 ± 44.0	218.3 ± 45.1	< .001
Triglycerides, mean (SD), mmol/l	155.6 ± 90.6	153.4 ± 89.6	163.1 ± 93.3	< .001
HDL cholesterol, mean (SD), mmol/l	56.1 ± 14.8	56.6 ± 14.8	54.3 ± 14.4	< .001
LDL cholesterol, mean (SD), mmol/l	137.9 ± 33.6	138.2 ± 33.5	136.8 ± 34.1	< .001
***Major chronic comorbidity***				
Hypercholesterolemia, No. (%)	69,494 (18.5%)	48,556 (16.8%)	20,938 (24.0%)	< .001
Hypertension, No. (%)	111,888 (29.7%)	81,520 (28.20%)	30,368 (34.9%)	< .001
Heart failure, No. (%)	2619 (0.7%)	1779 (0.6%)	840 (1.0%)	< .001
Chronic kidney disease, No. (%)	5738 (1.5%)	4110 (1.4%)	1628 (1.9%)	< .001
Any stroke, No. (%)	7948 (2.1%)	5439 (1.9%)	2509 (2.9%)	< .001
Diabetic hypoglycemia, No. (%)	2171 (0.6%)	1536 (0.5%)	635 (0.7%)	< .001
Type 2 diabetes mellitus, No. (%)	14,971 (4.2%)	10,487 (3.8%)	4484 (5.5%)	< .001
Coronary artery disease, No. (%)	21,576 (5.7%)	14,161 (4.9%)	7415 (8.5%)	< .001
***Medication***				
Use of ototoxic drugs, No. (%) (aspirin and/or ibuprofen consumption)	59,806 (22.5%)	44,584 (21.5%)	15,222 (25.9%)	< .001

*Abbreviations: ARHL* age-related hearing loss, *SD* standard deviation

^*^P-value indicates the significance of the difference between the control and ARHL case groups

For the replication set, a total of 36,357 PMBB participants of European (*n* = 26,523) and African American (*n* = 9834) descent were included (Additional file 1: Table S3). The mean age of participants was 55.7 years (SD, 16.4 years).

### PRS with ARHL and validation in the PMBB cohort

We used public large GWAS data to compute the PRS for ARHL and found the HL PRS to be robustly associated with ARHL prevalence (Additional file 1: Fig. S3). Table 2 presents the OR for ARHL association with PRS risk group. In the adjusted Model 1, we observed that individuals with a very high PRS had 1.58-fold increased risk of ARHL (95% CI, 1.47–1.70; *P* < 0.001). In the fully adjusted Model 4, individuals with a very high PRS had 1.49-fold increased risk of ARHL (95% CI, 1.36–1.62; *P* < 0.001), which remained significant.

**Table 2:** Odds ratio for ARHL associated with genetic risk group

				**Model 1**		**Model 2**		**Model 3**		**Model 4**	
Genetic risk	**Total no. of participants**	**No. of ARHL cases**	**Prevalence (%)**	**OR (95% CI)**	***P*-value**	**OR (95% CI)**	***P*-value**	**OR (95% CI)**	***P*-value**	**OR (95% CI)**	***P*-value**
**Low**	75,322	15,453	20.52	1 (reference)		1 (reference)		1 (reference)		1 (reference)	
**Intermediate**	225,879	52,060	23.05	1.16 (1.14–1.19)	< .001	1.15 (1.12–1.17)	< .001	1.14 (1.12–1.17)	< .001	1.14 (1.11–1.17)	< .001
**High**	71,499	18,477	25.84	1.36 (1.33–1.40)	< .001	1.35 (1.32–1.39)	< .001	1.34 (1.31–1.38)	< .001	1.34 (1.31–1.39)	< .001
**Very high**	3764	1076	28.59	1.58 (1.47–1.70)	< .001	1.55 (1.43–1.68)	< .001	1.49 (1.36–1.63)	< .001	1.49 (1.36–1.62)	< .001
**perSD increase**				1.12 (1.11–1.13)	< .001	1.12 (1.11–1.13)	< .001	1.12 (1.11–1.13)	< .001	1.12 (1.11–1.13)	< .001

Model 1 was adjusted by age, sex, genotype array, and PC 1 to 10

Model 2: Model 1 + education years + household income + Townsend deprivation index + number in household

Model 3: Model 2 + body mass index + height + weight + waist circumference + systolic blood pressure + diastolic blood pressure + Total cholesterol + HDL cholesterol + LDL cholesterol + Triglycerides + Creatinine + eGFR + Fasting blood glucose + HbA1c

Model 4: Model 3 + tinnitus + major chronic comorbidities (hypercholesterolemia, hypertension, heart failure, chronic kidney disease, any stroke, diabetic hypoglycemia, type 2 diabetes mellitus, and coronary artery disease)

*Abbreviations: ARHL* age-related hearing loss, *SD* standard deviation, *OR* odds ratio, *CI* confidence interval, *PC* principal component

We then replicated the PRS for ARHL in a cohort from the PMBB (Additional file 1: Table S3). We found a significant association between PRS and ARHL prevalence across ancestry in the overall PMBB cohort (Additional file 1: Table S4). The performance of each PRS based on the alternative methods (LDpred2, lassosum, and PRSice-2) is shown in Additional file 1: Table S5.

Individuals with a very high PRS had the highest ARHL OR of 2.22 (95% CI, 1.55–3.18; *P* < 0.001). In the PMBB dataset, sufficient environmental/lifestyle variables are not available, but relatively accurate disease onset information can be obtained. Therefore, we evaluated the association of PRS with ARHL occurrence using a Cox proportional hazard model. Again, higher PRS was significantly associated with increased HR for ARHL (low PRS, HR = 1 [reference]; intermediate PRS, HR = 1.10; high PRS, HR = 1.31; very high PRS, HR = 1.93; *P* < 0.001) (Additional file 1: Table S6). Individuals with a very high risk PRS showed a marked increase in the cumulative incidence of ARHL beginning at age 60 (Additional file 1: Fig. S4). Additionally, we calculated the incidence risk according to genetic risk across different age-at-onset groups. We observed a consistent increase in incidence risk with higher genetic risk across all age groups (Additional file 1: Table S7).

### Association of lifestyle/environmental factors with ARHL

In the adjusted Model, demographic data and lifestyle/environmental factors were associated with increased risk of ARHL (Additional file 1: Table S8 and Fig. S5). In particular, ARHL was highly associated with tinnitus frequency (OR, 6.39; 95% CI, 6.08–6.71), tinnitus severity (OR, 3.76; 95% CI, 3.29–4.29), time in a noisy workplace (OR, 2.57; 95% CI, 2.47–2.67), and loud music exposure frequency (OR, 2.09; 95% CI, 1.97–2.22). Additionally, we performed a multivariate regression analysis considering the mutual adjustment of lifestyle/environmental factors, and estimated the respective significances in Additional file 1: Table S9.

### Joint associations of HL PRS and lifestyle/environmental factors with ARHL

**Fig. 1:**
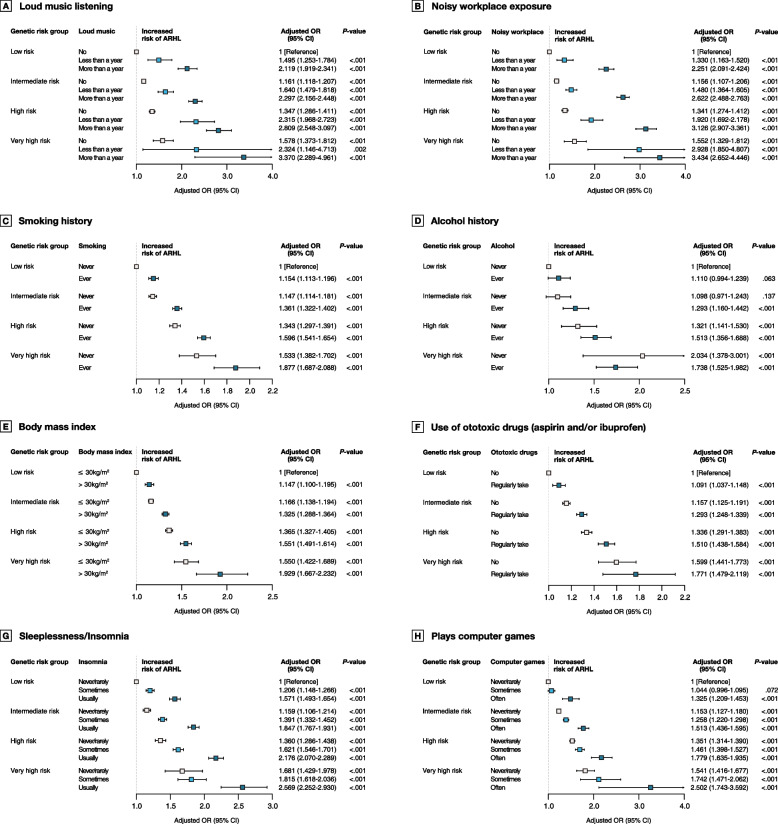
Odds ratio for ARHL according to genetic risk, lifestyle, and environmental factors. Model was adjusted by age, sex, genotype array, and PC 1 to 10. *P* for trends were significant in all analyses (*P* < .001). Abbreviations: ARHL, age-related hearing loss; OR, odds ratio; CI, confidence interval

To explore the effect of lifestyle/environmental factors on ARHL risk according to genetic risk, we stratified the lifestyle/environmental factors by PRS category (Fig. 1). We observed a monotonic association of increasing PRS and number of lifestyle/environmental factors with higher risk of ARHL. In particular, participants with very high PRS who worked in a noisy workplace for more than a year had the highest risk for ARHL (OR, 3.43; 95% CI, 2.65–4.45; *P* < 0.001), followed by those with very high PRS who listened to loud music for more than a year (OR, 3.37; 95% CI, 2.29–4.96; *P* < 0.001). We also confirmed that the risk for ARHL is high in groups for which a very high PRS is combined with the other unfavorable factors examined, except for alcohol history. The interactions were not significant (*P* > 0.05) in all lifestyle/environmental factors (Additional file 1: Table S10).

### Joint association of HL PRS and composite HLS on ARHL

In a fully adjusted Model 4, an ideal HLS based on selected lifestyle behaviors and environmental factors was significantly associated with lower risk of ARHL compared to very poor HLS (OR, 0.38; 95% CI, 0.33–0.43; *P* < 0.001) (Table 3 and Additional file 1: Table S11). We compared composite HLS with previously reported health-related scores. Firstly, AHA lifestyle score had a significant association (ideal, OR = 1[reference]; intermediate, OR = 1.06; poor, OR = 1.17) with risk of ARHL. MetS health score had a significant association (ideal, OR = 1; intermediate, OR = 1.17; poor, OR = 1.35) in the crude model, but its significance was limited after adjusting for baseline demographic information (age and sex). As a result, HLS showed a stronger association (ideal, OR = 1; intermediate, OR = 1.44; poor, OR = 2.11; very poor, OR = 3.03) for ARHL risk in the adjusted model compared to previous health-related scores (Additional file 1: Table S12).

**Table 3:** Odds ratio for ARHL associated with composite healthy lifestyle score

				**Model 1**		**Model 2**		**Model 3**		**Model 4**	
Lifestyle	**Total no. of participants**	**No. of ARHL cases**	**Prevalence (%)**	**OR (95% CI)**	***P*-value**	**OR (95% CI)**	***P*-value**	**OR (95% CI)**	***P*-value**	**OR (95% CI)**	***P*-value**
**Very poor**	3496	1303	37.27	1 (reference)		1 (reference)		1 (reference)		1 (reference)	
**Poor**	26,609	7652	28.76	0.69 (0.64–0.75)	< .001	0.70 (0.65–0.76)	< .001	0.68 (0.62–0.75)	< .001	0.74 (0.67–0.82)	< .001
**Intermediate**	49,094	10,064	20.50	0.48 (0.44–0.51)	< .001	0.48 (0.44–0.52)	< .001	0.46 (0.42–0.50)	< .001	0.53 (0.48–0.59)	< .001
**Ideal**	6389	936	14.65	0.33 (0.30–0.36)	< .001	0.32 (0.29–0.36)	< .001	0.30 (0.27–0.34)	< .001	0.38 (0.33–0.43)	< .001

Detailed information on the definition of the composite healthy lifestyle score is shown in Additional file 1: Method S7

Model 1 was adjusted by age, sex, genotype array, and PC 1 to 10

Model 2: Model 1 + education years + household income + Townsend deprivation index + number in household

Model 3: Model 2 + body mass index + height + weight + waist circumference + systolic blood pressure + diastolic blood pressure + Total cholesterol + HDL cholesterol + LDL cholesterol + Triglycerides + Creatinine + eGFR + Fasting blood glucose + HbA1c

Model 4: Model 3 + tinnitus + major chronic comorbidities (hypercholesterolemia, hypertension, heart failure, chronic kidney disease, any stroke, diabetic hypoglycemia, type 2 diabetes mellitus, and coronary artery disease)

*Abbreviations: ARHL* age-related hearing loss, *SD* standard deviation, *OR* odds ratio, *CI* confidence interval, *PC* principal component

**Fig. 2:**
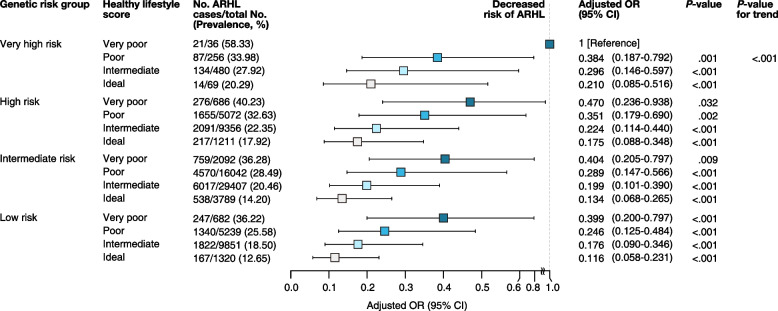
Forest plot for ARHL risk reduced by composite healthy lifestyle score in each genetic risk group. Model was adjusted by age, sex, genotype array, and PC 1 to 10. Abbreviations: ARHL, age-related hearing loss; OR, odds ratio; CI, confidence interval

We next stratified HLS by PRS using the group with very poor lifestyle and very high PRS as the reference. We found that the ideal lifestyle was associated with lower risk of ARHL in all genetic risk groups. In participants with very high genetic risk, an ideal lifestyle decreased the OR for ARHL to 0.21 (95% CI, 0.09–0.52; *P* < 0.001). Meanwhile, in participants with low genetic risk but very poor lifestyle, the OR for ARHL was 0.40 (95% CI, 0.20–0.80; *P* < 0.001) and those with low PRS and ideal lifestyle was 0.12 (95% CI, 0.06–0.23; *P* < 0.001) (Fig. 2).

### Stratification analysis according to sex and tinnitus history

The prevalence of ARHL was higher in males than in females (28.26% vs. 18.71%), but the degree to which OR increased in conjunction with increased PRS was higher in females (Additional file 1: Table S13). When stratifying according to tinnitus history, a similar association of increasing genetic risk and ARHL prevalence was found in those with tinnitus history relative to those without (Additional file 1: Table S14). However, there was no difference between groups when considering the joint association of ARHL incidence with HLS according to sex and tinnitus history (Additional file 1: Tables S15 and S16).

## Discussion

In this study, we demonstrated a joint association of genetic and lifestyle/environmental risk in influencing ARHL in a large-scale population of 376,464 UKBB participants. We also found that adherence to an ideal lifestyle in hearing-related respects could attenuate the risk of ARHL even in individuals with high genetic risk. To our knowledge, this is the first study to evaluate the joint effect among lifestyle/environmental risk factors and genetic risk and also adherence to healthy lifestyle on risk for ARHL.

Fransen et al. performed a GWAS including common and rare variants accounting for environmental factors, but found no variants that attained genome-wide significance for ARHL [12]. This finding suggests that the genetic structure of ARHL is highly polygenic, which structure is not readily explained by variants within major genes. Recently, a PRS calculated from self-reported HL in adults aged 40 to 69 years is associated with the hearing ability of children aged 11 to 12 years and also their parents [48]. This indicates that polygenic risk may play a role together with environmental risk factors in the development of ARHL. It has also been shown in an EHR-derived dataset that loss-of-function variants in known HL genes are strongly associated with risk for ARHL [49]. We demonstrated that the PRS for HL generated from the FinnGen dataset based on ICD code regarding the sensorineural HL could predict risk of ARHL in the UKBB dataset, which replicated in PMBB cohort. Our PRS for ARHL demonstrated a degree of predictive validity in the African American population, though it did not reach the level of significance observed in the European cohort. This discrepancy is likely attributable to the lack of non-European HL GWAS and the limited sample size in this study. Furthermore, we emphasize the need for careful consideration of approaches and linkage disequilibrium reference panels in PRS studies when estimating results across diverse populations. Future research necessitates enhanced data collection across different ethnicities and expanded cross-ancestry analysis to further validate and refine the predictive accuracy of the PRS for ARHL in diverse populations.

The effects of noise exposure on ARHL risk observed in this study are consistent with the results of previous studies [27]. Both noise-induced HL and ARHL are sensorineural HL resulting from dysfunction in the inner ear or cochlea, where sound-induced vibrations are converted by sensory hair cells into electrical signals; however, long-term prospective studies on the effect of continuous noise exposure on ARHL risk are lacking [50]. We further showed that not only noise exposure in the occupational environment but also loud music in daily life could be a significant risk factor for ARHL. Although the UKBB dataset did not provide precise information about noise exposures, we observed that more than 5 years of exposure to a noisy workplace or loud music was significantly associated with risk of ARHL.

While the effect of noise exposure on HL is well-established, it is still unclear whether there is a joint association between genetic risk and noise exposure. A mouse strain with ARHL is reportedly more sensitive to noise than other strains, suggesting a genetic predisposition to noise-induced HL in animals [51]. Fetoni et al. also suggested the connection between ARHL and noise exposure based on a mouse study, demonstrating that noise exposure at a young age accelerates and worsens ARHL phenotypes in mice due to damage in the cochlea [52]. Here, using a large population cohort, we showed that having genetic risk for HL significantly increases the risk of developing ARHL with increased exposure to noise in daily life. Accordingly, we could suggest that there is a significant association between genetic risk of ARHL and noise exposure.

Previous studies have consistently shown that smoking increases the risk of developing ARHL, but findings concerning the effect of alcohol have been mixed. In our results, smoking status clearly showed an additional risk for developing ARHL according to the ARHL PRS, whereas alcohol intake did not show a significant additional effect. In a cross-sectional study, alcohol intake was associated with decreased HL risk, whereas smoking status, including passive smoking, was associated with increased risk [18]. Fransen et al. also reported that smoking and high BMI increased ARHL risk and that moderate alcohol consumption had a protective role [23]. Meanwhile, several prospective studies have found no significant association between alcohol consumption and HL [28, 53, 54]. This discrepancy may be because different studies used different definitions of alcohol exposure, and more research is needed in the future.

Interestingly, when stratifying according to sex, the prevalence of ARHL was higher in males, but the degree to which greater PRS increased the odds of developing ARHL was higher in females. Epidemiological studies indicate that gender differences in ARHL prevalence cannot be attributed to differences in noise exposure [55]. Nolan et al. suggested that differences in cochlear physiology between females and males may exist from birth, so that hearing with aging may be modulated by sex [56]. The sex difference in ARHL risk according to genetic risk observed here can be explained by the Carter effect or gene-by-environment interaction. In particular, according to Carter’s model, the heritability of a trait may be higher in the sex with lower prevalence [57].

Among the many factors that lead to HL, exposure to noise, smoking, and ototoxic drugs can all be avoided through individual efforts as well as public health policies or clinical interventions. Using UKBB data, Yévenes-Briones et al. previously showed that a combination of healthy lifestyle behaviors is associated with lower risk of HL [17]; however, they did not consider genetic factors. Our study is the first to analyze the effect of adherence to healthy hearing-related lifestyle on ARHL risk according to genetic risk. We revealed that individuals with very high genetic risk for ARHL had an 80% reduction in odds when they maintained an ideal lifestyle. We also observed that ARHL genetic burden could be mostly overcome by lifestyle modification, suggesting that lifestyle modification is imperative for people with high ARHL PRS.

We first combined lifestyle/environmental factors and PRS to investigate the effect on and joint association with ARHL and found that a healthier lifestyle decreased risk much more significantly in individuals at high PRS percentiles. We then focused on the benefits of an integrated lifestyle in relation to genetic susceptibility to ARHL. Compared to previous modeling which considered environmental and genetic factors separately, this approach allows complex disease traits and multiple dimensions of lifestyle behavior to be better assessed.

## Limitations

Our study has some limitations. First, most of the lifestyle and environmental data was available only as of the time of the survey. Therefore, our study is a cross-sectional analysis rather than a prospective analysis of the effect of lifestyle on ARHL risk. Given this study design, it is difficult to infer a causal relationship between lifestyle and ARHL phenotype. Secondly, the population of this study consisted only of UKBB participants, who were aged 40 to 69 years at baseline and of European ancestry. We validated the PRS in an independent cohort and people of other ancestry, but the joint effect between genetic risk and lifestyle behavior has not been validated. Third, in this study, phenotyping of ARHL was based on a questionnaire. There are no definitive diagnostic criteria for ARHL, but accurate phenotyping to rule out other causes of HL may be necessary and requires evaluation by an otolaryngologist or audiometry. Finally, in our study, the HLS analysis was conducted on 85,588 individuals due to the presence of missing values in the factors. These individuals were different from the entire cohort, presenting with an older age, a higher proportion of males, and elevated income levels. Notably, these characteristics are in line with previous studies that have observed a tendency for cohorts with lower socioeconomic status to have higher rates of nonresponse answers in UKBB [58]. While the subset of individuals analyzed may not perfectly represent the entire UKBB cohort, the insights derived remain a valuable contribution to understanding the factors associated with ARHL.

## Conclusions

In conclusion, our findings demonstrate a joint effect between genetic risk and lifestyle/environmental factors in the development of ARHL. Furthermore, we found that an ideal lifestyle with regard to hearing is associated with reduced ARHL risk, even with genetic burden. Our results provided the evidence for clinicians to educate patients about the importance of behavioral modification for the prevention of ARHL. To demonstrate the clear benefits of modifying such risk factors in the prevention of ARHL, future prospective studies will be essential.

### Supplementary Information

**Additional file 1:** **Methods S1-11, Tables S1-16, and Figures S1-5.** **Method S1.** Penn Medicine Biobank banner author list and contribution statements. **Method S2.** Detailed definition of ARHL. **Method S3.** Detailed definitions of the covariates in the UK Biobank. **Method S4.** Detailed definitions of baseline major chronic comorbidities. **Method S5.** Detailed definitions of lifestyle factors, behaviors, and environmental factors in the UK Biobank. **Method S6.** Number of missing data for each variable in the UK Biobank. **Method S7.** Generating of composite healthy lifestyle score. **Method S8.** Detailed definitions of existing lifestyle score and metabolic syndrome status. **Method S9.** Detailed information on the genotype data quality control and imputation procedures. **Method S10.** Generating of polygenic risk score for ARHL. **Method S11.** Detailed information on statistical analysis. **Table S1.** Characteristics according to genetic risk group of ARHL in the UK Biobank; **Table S2.** Demographic comparison of the 85,588 participants included in the composite HLS analysis versus the remaining population within the UK Biobank. **Table S3.** Characteristics of participants in the Penn Medicine Biobank. **Table S4.** Odds ratio for ARHL associated with genetic risk group in the UK Biobank and Penn Medicine Biobank. **Table S5.** Proportion of the variance explained in ARHL by different PRS methods. **Table S6.** Cox proportional hazard model with age at ARHL onset in the Penn Medicine Biobank. **Table S7.** Incidence rates of ARHL according to HL PRS risk and age groups in the Penn Medicine Biobank. **Table S8.** Associations between lifestyle and environmental factors and ARHL. **Table S9.** Significance of each lifestyle/environmental factor in multivariate regression analysis considering mutual adjustments. **Table S10.** Significance of the interaction terms between each lifestyle/environmental factor and genetic risk group for ARHL. **Table S11.** Odds ratio for ARHL associated with healthy lifestyle score (Ideal lifestyle group as a reference). **Table S12.** Comparison between lifestyle scores and metabolic syndrome status (Ideal lifestyle group as a reference). **Table S13.** Odds ratio for ARHL according to genetic risk and sex. **Table S14.** Odds ratio for ARHL according to genetic risk and tinnitus history. **Table S15.** Odds ratio for ARHL according to Healthy lifestyle score and sex. **Table S16.** Odds ratio for ARHL according to Healthy lifestyle score and tinnitus history. **Fig. S1.** Study flowchart. **Fig. S2.** Flowchart for generating a composite healthy lifestyle score in the UK Biobank. **Fig. S3.** Density and prevalence plots according to genetic risk for ARHL distribution in the UK Biobank. **Fig. S4.** Cumulative incidence risk for onset age of ARHL in the Penn Medicine Biobank. **Fig. S5.** Correlation matrix of lifestyle and environmental factors associated with ARHL.

## Data Availability

The PRS model constructed in the current paper is available for download from the GitHub page (https://github.com/dokyoonkimlab/arhl-prs).

## Abbreviations

AHA: American Heart Association
ARHL: Age-related hearing loss
BMI: Body mass index
CI: Confidence interval
EHR: Electronic health record
GWAS: Genome-wide association study
HL: Hearing loss
HLS: Healthy lifestyle score
HR: Hazard ratio
ICD: International Classification of Diseases
MetS: Metabolic syndrome
OR: Odds ratio
PC: Principal component
PMBB: Penn Medicine Biobank
PRS: Polygenic risk score
QC: Quality control
SD: Standard deviation
SNP: Single-nucleotide polymorphism
UKBB: UK Biobank
