# Supplementary material for: Association between genetic risk and adherence to healthy lifestyle for developing age-related hearing loss
Source: BMC Med. 2024 Mar 26;22:141. doi: 10.1186/s12916-024-03364-5 (PMC10964689; doi:10.1186/s12916-024-03364-5)

Additional file 1.

Association between genetic risk and adherence to healthy lifestyle for developing age-related hearing loss. Sang-Hyuk Jung, Young Chan Lee, Manu Shivakumar, Jaeyoung Kim, Jae-Seung Yun, Woong-Yang Park, Hong-Hee Won, Penn Medicine Biobank, Dokyoon Kim.

Supplementary contents

Method S1. Penn Medicine Biobank banner author list and contribution statements.
Method S2. Detailed definition of ARHL.
Method S3. Detailed definitions of the covariates in the UK Biobank.
Method S4. Detailed definitions of baseline major chronic comorbidities.
Method S5. Detailed definitions of lifestyle factors, behaviors, and environmental factors in the UK Biobank.
Method S6. Number of missing data for each variable in the UK Biobank.
Method S7. Generating of composite healthy lifestyle score.
Method S8. Detailed definitions of existing lifestyle score and metabolic syndrome status.
Method S9. Detailed information on the genotype data quality control and imputation procedures.
Method S10. Generating of polygenic risk score for ARHL.
Method S11. Detailed information on statistical analysis.

Table S1. Characteristics according to genetic risk group of ARHL in the UK Biobank.
Table S2. Demographic comparison of the 85,588 participants included in the composite HLS analysis versus the remaining population within the UK Biobank.
Table S3. Characteristics of participants in the Penn Medicine Biobank.
Table S4. Odds ratio for ARHL associated with genetic risk group in the UK Biobank and Penn Medicine Biobank.
Table S5. Proportion of the variance explained in ARHL by different PRS methods.
Table S6. Cox proportional hazard model with age at ARHL onset in the Penn Medicine Biobank.
Table S7. Incidence rates of ARHL according to HL PRS risk and age groups in the Penn Medicine Biobank.
Table S8. Associations between lifestyle and environmental factors and ARHL.
Table S9. Significance of each lifestyle/environmental factor in multivariate regression analysis considering mutual adjustments.
Table S10. Significance of the interaction terms between each lifestyle/environmental factor and genetic risk group for ARHL.

Table S11. Odds ratio for ARHL associated with healthy lifestyle score (Ideal lifestyle group as a reference).
Table S12. Comparison between lifestyle scores and metabolic syndrome status (Ideal lifestyle group as a reference).
Table S13. Odds ratio for ARHL according to genetic risk and sex.
Table S14. Odds ratio for ARHL according to genetic risk and tinnitus history.
Table S15. Odds ratio for ARHL according to Healthy lifestyle score and sex.
Table S16. Odds ratio for ARHL according to Healthy lifestyle score and tinnitus history.

Figure S1. Study flowchart.
Figure S2. Flowchart for generating a composite healthy lifestyle score in the UK Biobank.
Figure S3. Density and prevalence plots according to genetic risk for ARHL distribution in the UK Biobank.
Figure S4. Cumulative incidence risk for onset age of ARHL in the Penn Medicine Biobank.
Figure S5. Correlation matrix of lifestyle and environmental factors associated with ARHL.

Method S1. Penn Medicine Biobank banner author list and contribution statements.

PMBB Leadership Team

Daniel J. Rader, M.D., Marylyn D. Ritchie, Ph.D.

Contribution: All authors contributed to securing funding, study design, and oversight. All authors reviewed the final version of the manuscript.

Patient Recruitment and Regulatory Oversight

JoEllen Weaver, Nawar Naseer, Ph.D., M.P.H., Giorgio Sirugo, M.D., Ph.D., Afiya Poindexter, Yi-An Ko, Ph.D., Kyle P. Nerz

Contributions: JW manages patient recruitment and regulatory oversight of study. NN manages participant engagement, assists with regulatory oversight, and researcher access. GS assists with researcher access. AP, YK, KPN perform recruitment and enrollment of study participants.

Lab Operations

JoEllen Weaver, Meghan Livingstone, Fred Vadivieso, Stephanie DerOhannessian, Teo Tran, Julia Stephanowski, Salma Santos, Ned Haubein, Ph.D., Joseph Dunn

Contribution: JW, ML, FV, SD conduct oversight of lab operations. ML, FV, AK, SD, TT, JS, SS perform sample processing. NH, JD are responsible for sample tracking and the laboratory information management system.

Clinical Informatics

Anurag Verma, Ph.D., Colleen Morse Kripke, M.S. DPT, MSA, Marjorie Risman, M.S., Renae Judy, B.S., Colin Wollack, M.S.

Contribution: All authors contributed to the development and validation of clinical phenotypes used to identify study subjects and (when applicable) controls.

Genome Informatics

Anurag Verma Ph.D., Shefali S. Verma, Ph.D., Scott Damrauer, M.D., Yuki Bradford, M.S., Scott Dudek, M.S., Theodore Drivas, M.D., Ph.D.

Contribution: AV, SSV, and SD are responsible for the analysis, design, and infrastructure needed to quality control genotype and exome data. YB performs the analysis. TD and AV provide variants and gene annotations and their functional interpretation of variants.

Method S2. Detailed definition of ARHL.

Study reference	Definition	Detail criteria[§]		
		ARHL case	Control	Exclusion
**UK Biobank** (Validation set in this study)	Age-related hearing loss	If the participants answered 'Yes' to Question 3 or 'Yes' to both Questions 1 when they were aged ≥ 40 years.	Participants that answered 'No' to all these questions were classified as control.	Conductive hearing loss and congenital disorder that causes impairment of hearing (ICD-10: H90.0, H90.1, H90.2, H91.3, Q16.1, Q16.3, Q16.4, Q16.5, or Q16.9) or who reported being completely deaf.
**Penn Medicine Biobank** (Replication set in this study)	Age-related hearing loss	Sensorineural hearing loss (ICD-9: 389.1, 388.01; ICD-10: H90.3, H90.4, H90.5, or H91.1) with aged ≥ 40 years.	Participants without any hearing-related disease.	Conductive hearing loss and congenital disorder that causes impairment of hearing (ICD-10: H90.0, H90.1, H90.2, H91.3, Q16.1, Q16.3, Q16.4, Q16.5, or Q16.9)
Hoffmann et al. 2016 [11]	Age-related hearing impairment	If answered "yes" to both Questions 1 and 2.	If answered "no" to both questions.	Participants who reported being completely deaf.
Lewis et al. 2022 [23]	Hearing difficulty in older adults	If answered "yes" to Question 1 or 3 when they were aged ≥ 55 years.	If answered "no" to both Questions 1 and 3 when they were aged ≥ 55 years.	n/a
Liu et al. 2021 [24]	Age-related hearing loss	If answered "yes" to both Question 1 or 3.	n/a	n/a
Wells et al. 2019 [25]	1) self-reported hearing difficulty<br>2) self-reported hearing aid use	1) If answered "yes" to Question 1<br>2) If answered "yes" to Question 3	If answered "no" to both Questions 1 and 3 with aged ≥50 years.	n/a

[§]Questionnaires

Question 1: Do you have any difficulty with your hearing? [UK Biobank field ID: 2247]

Question 2: Do you find it difficult to follow a conversation if there is background noise (such as TV, radio, or children playing)? [UK Biobank field ID: 2257]

Question 3: Do you use a hearing aid most of the time? [UK Biobank field ID: 3393]

Method S3. Detailed definitions of the covariates in the UK Biobank.

During the enrollment process, participants provided information on their sociodemographic characteristics, health and medical history, and lifestyle factors through a self-administered touchscreen questionnaire and in-person baseline interviews. Additionally, trained staff measured the height, body weight, waist circumference, and hip circumference of participants during the interviews using standardized procedures. Educational level qualifications were coded by mapping the highest level of education that a respondent achieved to an International Standard Classification of Education 1997 category and then imputing a years-of-education equivalent for each International Standard Classification of Education 1997 category [21]. Household income before tax was divided into five groups: less than £18 000, £18 000 to £30 999, £31 000 to £51 999, £52 000 to £100 000, and greater than £100 000 (exchange rate in May 2023 of $1.00 = £0.79).

Blood samples were collected during the baseline assessment visit, and all procedures for sampling and processing of blood and urine samples were performed using standardized protocols [26]. To measure HbA1c, high performance liquid chromatography was utilized with the Bio-Rad Variant II Turbo Analyzer. Additionally, the Beckman Coulter AU5800 was used to determine glucose, lipid profiles, and inflammatory markers. Hexokinase analysis was employed for HbA1c, CHO-POD analysis for total cholesterol, GPO-POD analysis for triglycerides, enzyme immunoinhibition analysis for high-density lipoprotein (HDL) cholesterol, enzymatic selective protection analysis for low-density lipoprotein (LDL) cholesterol, and immunoturbidimetric assays for lipoprotein (a) and high sensitivity C-reactive protein. These assays provided accurate and precise measurements of the various biomarkers. Further details regarding serum biomarker data are available on the UK Biobank website at https://www.ukbiobank.ac.uk, where interested

parties can find more information about the data collection process, data quality control, and any limitations associated with the data.

Information regarding major chronic comorbidities was obtained from (1) the self-report collected via in-person interview or touchscreen questionnaire at enrollment, (2) diagnostic or procedure codes in the electronic health records database linked to hospital admission records, and (3) the first occurrence of the comorbidity in the health outcomes database, which is linked with hospital in-patient records, death records, cancer registry, and primary care records. The history of major chronic comorbidity or tinnitus were coded as binary variables. Detailed criteria are described in **Method S4.**

Method S4. Detailed definitions of baseline major chronic comorbidities.

Comorbidity	Path	Field ID	Code
Baseline hypercholesterolaemia	Verbal interview	Non-cancer illness, self-report (20002)	High cholesterol (1473)
	First occurrence before enrollment	First reported of disorders of lipoprotein metabolism and other lipidemia (130815, 130816)	E78.x
	Medication	Medication for cholesterol, blood pressure or diabetes (6177)	Cholesterol lowering medication
Baseline hypertension	Verbal interview	Non-cancer illness, self-report (20002)	1065, 1072
	Touchscreen	Vascular/heart problems diagnosed by doctor (6150)	High blood pressure
	First occurrence before enrollment	First reported of essential hypertension (131286, 131287)	I10.x
		First reported of hypertensive heart disease (131288, 131289)	I11.x
		First reported of hypertensive renal disease (131290, 131291)	I12.x
		First reported of hypertensive heart and renal disease (131292, 131293)	I13.x
		First reported of secondary hypertension (131294, 131295)	I15.x
	Medication	Medication for cholesterol, blood pressure or diabetes (6177)	Blood pressure medication
Baseline type 2 diabetes mellitus	Verbal interview	Non-cancer illness, self-report (20002)	Diabetes (1220) Type 2 diabetes (1223)
	Touchscreen	Diabetes diagnosed by doctor (2443)	Yes
	First occurrence before enrollment	First reported of non-insulin-dependent diabetes mellitus (130708, 130709)	E11.x
		First reported of unspecified diabetes mellitus (130714, 130715)	E14.x
	Medication	Treatment/medication code (20003)	Insulin (1140883066) Metformin (1140884600, 1141189090) Sulfonylurea (1141152590, 1140874744, 1140874718, 1141156984) Acarbose (1140868902) Thiazolidinedione (1141171646) Meglitinide (1141168660, 1141173882)
	HbA1c at baseline	Glycated hemoglobin (HbA1c) (30750)	≥6.5%
	Verbal interview for exclusion type 1 diabetes	Non-cancer illness, self-report (20002)	Type 1 diabetes (1222)

	First occurrence for exclusion type 1 diabetes	First reported of insulin-dependent diabetes mellitus (130706, 130707)	E10.x
Baseline coronary artery disease/myocardial infarction	Verbal interview	Non-cancer illness, self-report (20002)	1074 (angina)<br>1075 (heart attack/myocardial infarction)
	Touchscreen	Vascular/heart problems diagnosed by doctor (6150)	Heart attack, angina
	First occurrence before enrollment	First reported of angina pectoris (131296, 131297)	I20.x
		First reported of acute myocardial infarction (131298, 131299)	I21.x
		First reported of subsequent myocardial infarction (131300, 131301)	I22.x
		First reported of certain current complications following acute myocardial infarction (131302, 131303)	I23.x
		First reported of other acute ischemic heart diseases (131304, 131305)	I24.x
		First reported of chronic ischemic heart disease (131306, 131307)	I25.x
Baseline heart failure	Verbal interview	Non-cancer illness, self-report (20002)	Heart failure (1076, 1079)
	Hospital inpatient data	Summary Diagnosis (ICD10, 41270)	I50, I50.0, I50.1, I50.9
		Summary Diagnosis (ICD9, 41271)	4254, 4280, 4281, 4289
Baseline ischemic stroke	Verbal interview	Non-cancer illness, self-report (20002)	Stroke (1081,1082,1583)
	First occurrence before enrollment	First reported of cerebral infarction (131366, 131367)	I63.x
Baseline hemorrhagic stroke	Verbal interview	Non-cancer illness, self-report (20002)	Stroke (1081)
	First occurrence before enrollment	First reported of subarachnoid hemorrhage (131360, 131361)	I60.x
		First reported of intracerebral hemorrhage (131362, 131363)	I61.x
		First reported of other nontraumatic intracranial hemorrhage (131364, 131365)	I62.x
Baseline chronic kidney disease	Verbal interview	Non-cancer illness, self-report (20002)	Renal/kidney failure (1192)<br>Renal failure requiring dialysis (1193)<br>Renal failure not requiring dialysis (1194)
	First occurrence before enrollment	First reported of chronic renal failure (132030, 132031)	N18.x
		First reported of unspecified renal failure (132032, 132033)	N19.x

Method S5. Detailed definitions of lifestyle factors, behaviors, and environmental factors in the UK Biobank.

Factor	Question or Description	Field ID
**Environment factor**		
Noisy workplace	Have you ever worked in a noisy place where you had to shout to be heard?	4825
Workplace very noisy	Thinking about the place where you worked: Was it very noisy?	22606
Daytime sound level of noise pollution, dB	LDay (day equivalent level): Average sound level pressure LAeq over the 12-hour period 07:00 to 19:00.	24020
Evening sound level of noise pollution, dB	LEve (evening equivalent level): Average sound level pressure LAeq between the hours of 19:00 to 23:00.	24021
Night-time sound level of noise pollution, dB	LNight (night equivalent level): Average sound level pressure LAeq overnight 23:00 to 07:00.	24022
16-hour sound level of noise pollution, dB	LAeq,16hr (A-weighted equivalent sound level): Average sound level pressure LAeq between the hours of 07:00 to 23:00.	24023
24-hour sound level of noise pollution, dB	LDen: (day-evening-night equivalent level): A-weighted Leq noise level measured over the 24 hour period with a 10 decibel penality added to the levels between 23:00 and 07:00.	24024
**Lifestyle factor**		
Obesity	Body mass index at baseline	21001
Loud music exposure frequency	Have you ever listened to music for more than 3 hours per week at a volume which you would need to shout to be heard or, if wearing headphones, someone else would need to shout for you to hear them?	4836
Time spent watching television (TV)	In a typical DAY, how many hours do you spend watching TV?	1070
Time spent using computer	In a typical DAY, how many hours do you spend using the computer?	1080
Length of mobile phone use	For approximately how many years have you been using a mobile phone at least once per week to make or receive calls?	1110
Plays computer games	Do you play computer games?	2237
Number of days/week walked 10+ minutes	In a typical WEEK, on how many days did you walk for at least 10 minutes at a time?	864
Number of days/week of moderate physical activity 10+ minutes	In a typical WEEK, on how many days did you do 10 minutes or more of moderate physical activities like carrying light loads, cycling at normal pace?	884
Usual walking pace	How would you describe your usual walking pace?	924
Sleep duration	About how many hours sleep do you get in every 24 hours? (Please include naps)	1160
Nap during day	Do you have a nap during the day?	1190
Sleeplessness / insomnia	Do you have trouble falling asleep at night or do you wake up in the middle of the night?	1200
Alcohol drinker status	Never / Ever	20117
Smoking status	Never / Ever	20116
Use of ototoxic drugs	Do you regularly take any of the following? (Specially aspirin and/or ibuprofen consumption)	6154

Method S6. Number of missing data for each variable in the UK Biobank.

Variable	**No. of missing (%)** (No. of total: 376,464)
Education years	3,198 (0.85%)
Number in household	2,022 (0.54%)
Townsend deprivation index	507 (0.13%)
Average total household income before tax	52,392 (13.92%)
Body composition	
Body mass index	1,176 (0.31%)
Height	795 (0.21%)
Weight	1,042 (0.28%)
Waist circumference	614 (0.16%)
Systolic blood pressure, mean (SD), mmHg	337 (0.09%)
Diastolic blood pressure, mean (SD), mmHg	335 (0.09%)
Hearing condition	
Speech reception threshold	223,967 (59.49%)
Tinnitus	254,802 (67.68%)
Tinnitus severity/nuisance	341,231 (90.64%)
Environmental factor	
Workplace very noisy	282,241 (74.97%)
Noisy workplace	253,668 (67.38%)
Average daytime sound level of noise pollution (dB)	7,375 (1.96%)
Average evening sound level of noise pollution (dB)	7,375 (1.96%)
Average night-time sound level of noise pollution (dB)	7,375 (1.96%)
Average 16-hour sound level of noise pollution (dB)	7,375 (1.96%)
Average 24-hour sound level of noise pollution (dB)	7,375 (1.96%)
Lifestyle factor	
Loud music exposure frequency	254,258 (67.54%)
Plays computer games	189 (0.05%)
Sleeplessness/insomnia	290 (0.08%)
Alcohol intake frequency (categorical)	57 (0.02%)
Laboratory result	
Total cholesterol, mean (SD), mmol/l	17,508 (4.65%)
Triglycerides, mean (SD), mmol/l	17,799 (4.73%)
HDL cholesterol, mean (SD), mmol/l	47,997 (12.75%)
LDL cholesterol, mean (SD), mmol/l	18,172 (4.83%)
Major chronic comorbidity	
Heart failure	11 (0.00%)
Type 2 diabetes mellitus	21,289 (5.65%)
Medications	
Use of ototoxic drug (aspirin and ibuprofen consumption)	110,645 (29.39%)

Method S7. Generating of Healthy lifestyle score in the UK Biobank.

	Factor	Question or Description	Healthy lifestyle status	Field ID
1	Noisy workplace	Have you ever worked in a noisy place where you had to shout to be heard?	No (expose)	4825
2	Obesity	BMI at baseline	<30 kg/m^2	21001
3	Loud music exposure frequency	Have you ever listened to music for more than 3 hours per week at a volume which you would need to shout to be heard or, if wearing headphones, someone else would need to shout for you to hear them?	No	4836
4	Plays computer games	Do you play computer games?	Rarely/never	2237
5	Sleeplessness / insomnia	Do you have trouble falling asleep at night or do you wake up in the middle of the night?	Rarely/never	1200
6	Alcohol drinker status	Never/Ever	Never	20117
7	Smoking status	Never/Ever	Never	20116
8	Use of ototoxic drugs	Do you regularly take any of the following? (Specially aspirin and/or ibuprofen consumption)	No	6154

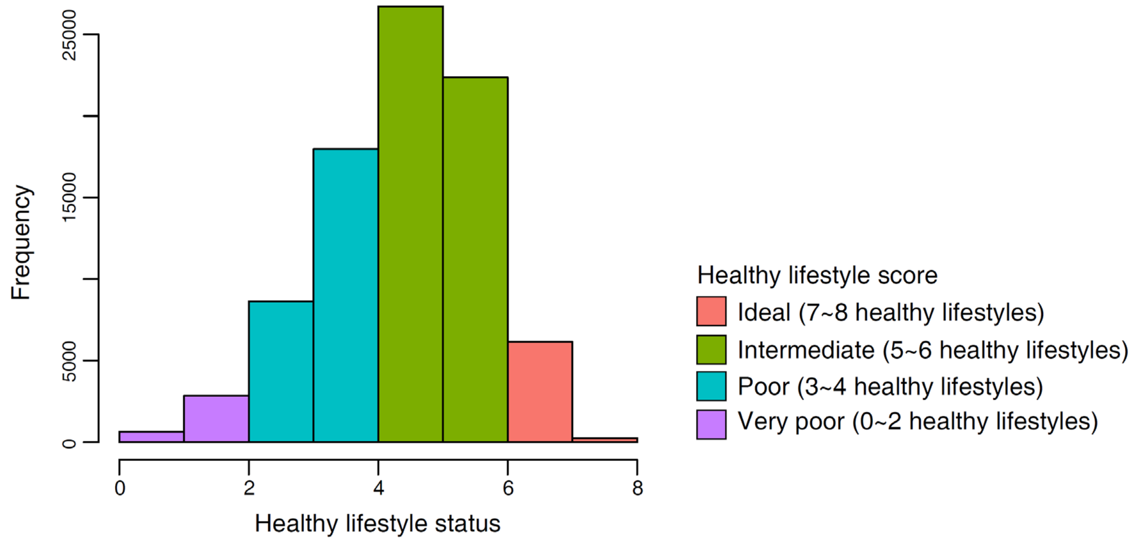

**Healthy lifestyle score**	**Ideal**		**Intermediate**		**Poor**		**Very poor**		
**No. of samples**	**6,389**		**49,094**		**26,609**		**3,496**		
No. of healthy lifestyle status	8	7	6	5	4	3	2	1	0
No. of samples	233	6,156	22,364	26730	17,975	8,634	2,857	582	57

Method S8. Detailed definitions of existing lifestyle score and metabolic syndrome status.

**Lifestyle factors**	**Component**	**Healthy lifestyle or metabolic status**	**Field ID of UK biobank**
Current smoking	Current smoking at baseline	Absence	20116
Alcohol consumption	Alcohol consumption at baseline	Never or moderate consumption: women: 0 and ≤14g/day men: 0 and ≤28g/day or never deinked	1558 (Never), 20117 (Never), 26030
Obesity	BMI at baseline	<30 kg/m^2	21001
Physical activity	Number of days per week of physical activity 10+ minutes	Participating in moderate activity ≥5 days a week or vigorous activity ≥3 days a week	884 (Moderate physical activity 10+ minutes)<br>904 (Vigorous physical activity 10+ minutes)
Eating habits	At least half of all following diet components were considered as a healthy lifestyle, less than half were considered as an unhealthy lifestyle		
	Fruit	≥3 serving/day	1309 (Fresh fruit)<br>1319 (Dried fruit)
	Vegetable	≥3 serving/day	1289 (Cooked Vegetable)<br>1299 (Salad or raw Vegetable)
	Whole grains	≥3 serving/day	1438, 1448 (Wholemeal or wholegrain bread)<br>1458, 1468 (Bran, oat, muesli cereal)
	Fish	≥2 serving/week	1329 (Oily fish)<br>1339 (Non-oily fish)
	Dairy	≥2.5 serving/week	1408 (Cheese)<br>1418 (Milk)
	Refined grains	≤1.5 serving/week	1438, 1448 (Wholemeal or wholegrain bread)<br>1458, 1468 (Bran, oat, muesli cereal)
	Processed meats	≤1 serving/week	1349 (Processed meat)
	Unprocessed meats	≤1.5 serving/week	1359 (Poultry)<br>1369 (Beef)<br>1379 (Lamb)<br>1389 (Pork)
**Lifestyle behavior**	Favorable	Having at least three healthy lifestyle factors	
	Intermediate	Having two healthy lifestyle factors	
	Unfavorable	Having one or fewer healthy lifestyle factor	

**Metabolic syndrome**	**Component**	**Healthy lifestyle or metabolic status**	**Field ID of UK biobank**
	Waist circumference	Men <102 cm<br>Women <88 cm	48
	Triglyceride	<1.70 mmol/L	30870
	HDL cholesterol	≥1.03 mmol/L	30760
	Glucose (or having diabetes)	≥5.6 mmol/L	30740
	Systolic or diastolic blood pressure	≥130 mmHg or<br>≥ 85 mmHg	4080<br>4079

Method S9. Detailed information on the genotype data quality control and imputation procedures.

UK Biobank

UK Biobank samples (version 3; March 2018) were genotyped for > 800,000 SNPs using either the Affymetrix UK BiLEVE Axiom array or the Affymetrix UK Biobank Axiom array. Imputation was carried out centrally by UK Biobank researchers using the merged 1000 Genomes Project panel and UK 10K panel; SHAPEIT3 was used for phasing and IMPUTE2 was used for imputation (GRCh37/hg19) [33, 34]. After imputation, variant-level quality control (QC) was performed by filtering SNPs on two criteria: (i) minor allele frequency < 1%, (ii) imputation quality score (INFO) < 0.3, and (iii) the Hardy–Weinberg equilibrium with a *P*-value of < 10^{-6}. A total of 9,505,768 imputed autosomal SNPs passed the QC criteria. Sample-level QC was performed by excluding samples on the basis of (i) participants identified as not of 'White-British' ancestry according to either self-report or principal components (PC) analysis of genetic ancestry and (ii) mismatched sex. After exclusion, 376,464 White-British participants were determined eligible for the genetic analyses.

Penn Medicine Biobank

Penn Medicine Biobank consists of 43,623 samples that have been genotyped by the GSA genotyping array. We performed genotype imputation for the Penn Medicine Biobank dataset using Eagle2 [35] and Minimac4 [36] softwares on TOPMed Imputation Server [37]. Imputation was performed for all autosomes, with TOPMed version R2 on the GRCh38 reference panel [38]. After imputation, variant-level QC was performed by filtering SNPs on three criteria: 1) minor allele frequency <0.01, 2) marker call rate <0.05, and 3) INFO <0.2. Sample-level QC was performed by excluding samples on the basis of 1) mismatched sex or 2) having second-degree or

closer relatives also in the Biobank. We inferred ancestry by projecting array genotype data onto PC axes defined by individuals from the 1000 Genome Project [39]. After exclusion, a total of 26,523 individuals considered European (non-Hispanic White) ancestry and 9,834 individuals considered African American (non-Hispanic Black) ancestry were determined eligible for the replication analyses.

Method S10. Generating of polygenic risk score for ARHL.

To generate polygenic risk score (PRS), we utilized the HL-GWAS summary statistics from the FinnGen Consortium (Data Freeze R8v4) which is a large-scale public-private partnership combining digital health record data from Finnish health registries with genotyping data from Finnish Biobanks [40]. FinnGen Release 8 (https://www.finngen.fi/en) includes association data at 20,175,454 variants for 2,202 endpoints in 342,499 (190,879 females and 151,620 males) Finnish individuals. The sensorineural HL cases (H8_HL_SEN_NAS) were identified based on ICD codes (ICD-9: 3891; ICD-10: H90.3, H90.4, and H90.5). There were 28,310 cases and 302,750 controls following the FinnGen phenotype definition. GWAS was performed by FinnGen using SAIGE with sex, age, 10 PCs, and genotyping batch as covariates [43]. In addition, we mapped FinnGen summary statistics from GRCh38 to GRCh37/hg19 using the liftOver tool to perform PRS with UK Biobank genotyped data (GRCh37/hg19) [44].

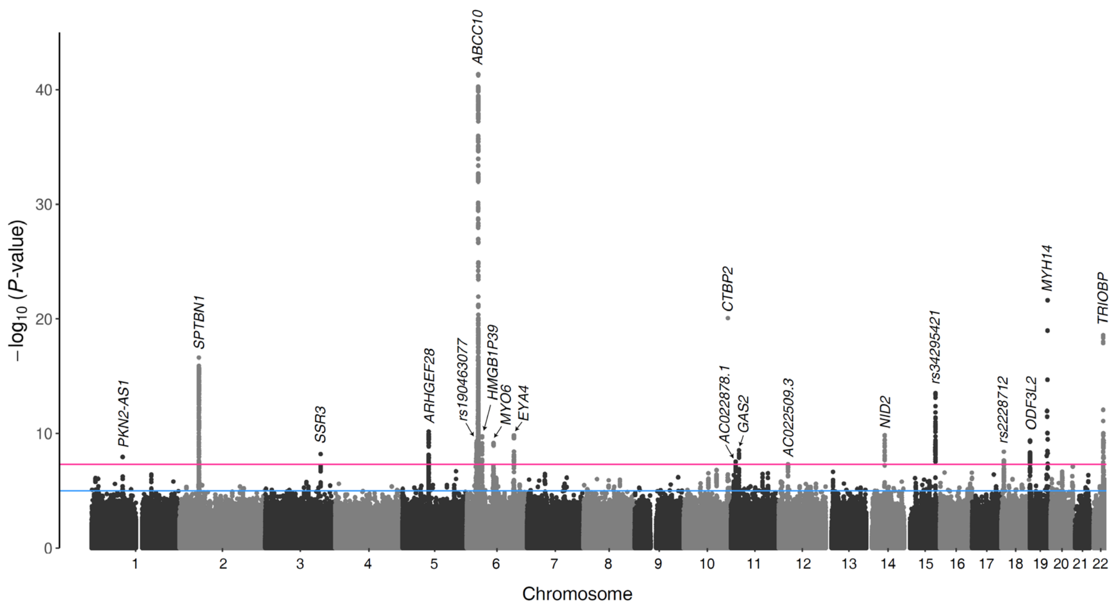

Figure. Manhattan plot for the sensorineural hearing loss GWAS derived from the FINNGEN release version 8.

We constructed PRS for HL by using a Bayesian polygenic prediction method, PRS-CS [41], which infers the posterior mean effect size of each variant using the linkage disequilibrium (LD) reference panel and GWAS summary. The 1000G Project phase 3 EUR data was used to be the external LD reference panel. The posterior SNP effect sizes in PRS-CS were inferred from GWAS summary statistics for EUR (FinnGen r8) population. The individual PRSs were computed from beta coefficients as the weighted sum of the risk alleles by applying PLINK version 1.90 with the --score command [42].

To compare other PRS methods, we generated PRSs using several alternative methods, including LDpred2 [45], lassosum [46], and PRSice-2 [47]. (i) For LDpred2 algorithm, a Bayesian method to derive PRSs using information on genetic architecture (SNP-based heritability and polygenicity measured as the fraction of causal variants) and on LD obtained from a 1000G Project reference panel, the variants were restricted to HapMap3 SNPs as recommended, we ran the LDpred2-auto model (‘bigsnpr’ R package v1.12.2) with the parameters: *p* (proportion of causal variants) in a sequence of five values from 10^{-4} to 1 on a log-scale and sparse option (true or false); (ii) For lassosum, a penalized regression method that accounts for LD, we used default parameters (shrinkage parameter: 0.2, 0.5, 0.9 and 1; penalty parameter (λ): varied from 0.001 to 0.1) and a random sample of 10,000 European ancestry individuals from the target set (UK Biobank) as a reference panel; (iii) For PRSice-2 (the pruning and thresholding method) software, we applied the *P*-value thresholds as 1, 5.0×10^{-2}, 1.0×10^{-2}, 1.0×10^{-4}, 1.0×10^{-5}, 1.0×10^{-6}, and 5.0×10^{-8} (in a window size 250kb), and the r2 thresholds as 0.1, 0.5 and 0.9.

The performance of the PRS was measured as Nagelkerke’s pseudo-R2 obtained by normalized PRS.

Method S11. Detailed information on statistical analysis.

The demographic and clinical characteristics were presented as mean ± standard deviation (SD) or numbers (percentage). Continuous variables were compared by the student's t-test or Mann-Whitney U test as appropriate. Categorical variables were compared by the chi-square test or Fisher's exact test, as appropriate.

We used a multivariate logistic regression model to evaluate the association of HL-PRS, and lifestyle/environmental factors with ARHL. In the primary analysis, we calculated odds ratio (OR) and 95% confidence intervals (CI) after adjusting for age, sex, the first ten principal components (PCs) of ancestry, and genotyping array type in the multivariate logistic regression model. The ORs of PRSs for ARHL were used both as quantitative variables reported per one-SD and categorical variables defined as follows: low (<20th percentile), intermediate (20–80th percentile), high (80–99th percentile), and very high (>99th percentile). In the sensitivity analyses, regression models were additionally adjusted for Townsend deprivation index, income level, number in household, education year, baseline blood pressure, laboratory findings, tinnitus, and major chronic comorbidities (including hypercholesterolaemia, hypertension, heart failure, chronic kidney disease, any stroke, diabetic hypoglycemia, type 2 diabetes mellitus, and coronary artery disease). Subsequently, we conducted joint association analyses to investigate the interplay between genetic and lifestyle/environmental factors. An interaction term in the regression model was included to test for statistical interaction between lifestyle/environmental factors, and genetic risk in relation to ARHL. In additional, we performed a stratified analysis according to sex and history of tinnitus.

To evaluate whether the participants with a high PRS showed earlier development of ARHL than did those with a low PRS, we performed a Cox proportional hazards regression

analysis with age at ARHL onset and age at the last clinical visit as time variables and the diagnosis of ARHL as a status and calculated Hazards ratio (HR) and 95% CIs in the Penn Medicine Biobank. Kaplan-Meier curves were conducted to check if there were significant differences in survival between genetic risk groups.

All statistical tests were two-sided, and $P < 0.05$ was considered statistically significant. All statistical analyses were conducted using R Statistical Software (version 4.1.0; R Foundation for Statistical Computing, Vienna, Austria) and PLINK version 1.90 [44].

Table S1. Characteristics according to genetic risk group of ARHL in the UK Biobank.

	Low genetic risk group (n=75,322)	Intermediate genetic risk group (n=225,879)	High genetic risk group (n=71,499)	Very high genetic risk group (n=3,764)	*P*-value
Age, mean (SD), y	57.5 ± 7.9	57.5 ± 7.9	57.5 ± 7.9	57.4 ± 8.0	.456
Sex, No. (%)					.858
Male	34,760 (46.1%)	104,552 (46.3%)	33,142 (46.4%)	1,751 (46.5%)	
Female	40,562 (53.9%)	121,327 (53.7%)	38,357 (53.6%)	2,013 (53.5%)	
Education years, mean (SD), y	13.8 ± 5.1	13.8 ± 5.1	13.8 ± 5.1	13.7 ± 5.1	.328
Number in household, mean (SD)	2.4 ± 1.2	2.4 ± 1.3	2.4 ± 1.2	2.4 ± 1.2	.603
Townsend deprivation index, mean (SD)	-1.6 ± 2.9	-1.6 ± 2.9	-1.6 ± 2.9	-1.6 ± 2.9	.586
Average total household income before tax					.170
Less than £18,000	14,211 (21.9%)	43,034 (22.1%)	13,612 (22.1%)	740 (22.9%)	
1,8000 to 30,999£	16,576 (25.5%)	50,269 (25.9%)	16,026 (26.0%)	827 (25.6%)	
31,000 to 51,999£	17,356 (26.7%)	51,098 (26.3%)	16,243 (26.4%)	857 (26.6%)	
52,000 to 100,000£	13,258 (20.4%)	39,719 (20.4%)	12,610 (20.5%)	642 (19.9%)	
Greater than 100,000£	3,502 (5.4%)	10,206 (5.3%)	3,126 (5.1%)	160 (5.0%)	
Body composition					
Body mass index, mean (SD), kg/m^2	27.4 ± 4.7	27.4 ± 4.8	27.4 ± 4.8	27.5 ± 4.8	<.001
Height, mean (SD), cm	82.5 ± 10.7	82.3 ± 10.7	82.2 ± 10.6	82.0 ± 10.8	<.001
Weight, mean (SD), kg	78.2 ± 15.9	78.4 ± 15.9	78.4 ± 15.9	78.3 ± 15.9	.026
Waist circumference, mean (SD), cm	90.2 ± 13.5	90.4 ± 13.5	90.5 ± 13.4	90.6 ± 13.7	.002
Systolic blood pressure, mean (SD), mmHg	140.5 ± 19.7	140.3 ± 19.7	140.0 ± 19.5	139.8 ± 19.8	<.001
Diastolic blood pressure, mean (SD), mmHg	82.5 ± 10.7	82.3 ± 10.7	82.2 ± 10.6	82.0 ± 10.8	<.001
Hearing condition					
Speech reception threshold (SRT), No. (%)					.001
Normal (SRT < −5.5 dB)	24,074 (79.7%)	72,663 (79.2%)	22,741 (78.4%)	1,198 (78.6%)	
Insufficient (−5.5 dB to −3.5 dB)	4,992 (16.5%)	15,304 (16.7%)	5,009 (17.3%)	254 (16.7%)	
Poor (SRT > −3.5 dB)	1,151 (3.8%)	3,767 (4.1%)	1,271 (4.4%)	73 (4.8%)	
Tinnitus, No. (%)					<.001
No, never	17,418 (72.3%)	51,753 (70.8%)	16,169 (69.5%)	841 (69.0%)	
Yes, but not now, but have in the past	2,634 (10.9%)	8,082 (11.1%)	2,543 (10.9%)	123 (10.1%)	
Yes, now some of the time	2,030 (8.4%)	6,554 (9.0%)	2,218 (9.5%)	111 (9.1%)	
Yes, now a lot of the time	548 (2.3%)	1,930 (2.6%)	592 (2.5%)	44 (3.6%)	
Yes, now most or all of the time	1,457 (6.0%)	4,772 (6.5%)	1,743 (7.5%)	100 (8.2%)	
Tinnitus severity/nuisance, No. (%)					.209
Not at all	2,170 (32.8%)	6,802 (32.1%)	2,323 (33.0%)	117 (31.0%)	
Slightly	3,167 (47.8%)	10,300 (48.6%)	3,337 (47.3%)	176 (46.7%)	
Moderately	1,069 (16.2%)	3,462 (16.3%)	1,158 (16.4%)	77 (20.4%)	
Severely	213 (3.2%)	625 (2.9%)	230 (3.3%)	7 (1.9%)	
Environmental factor					
Noisy workplace, No. (%)					.772
No	18,631 (76.6%)	56,343 (76.4%)	17,888 (76.2%)	917 (74.3%)	
Yes, for less than a year	1,308 (5.4%)	4,026 (5.5%)	1,275 (5.4%)	72 (5.8%)	
Yes, for around 1-5 years	1,366 (5.6%)	4,234 (5.7%)	1,392 (5.9%)	76 (6.2%)	
Yes, for more than 5 years	3,004 (12.4%)	9,182 (12.4%)	2,913 (12.4%)	169 (13.7%)	
Workplace very noisy, No. (%)					.288
Rarely/never	10,314 (55.1%)	30,931 (54.9%)	9,867 (54.4%)	545 (54.3%)	
Sometimes	6,587 (35.2%)	19,711 (35.0%)	6,433 (35.5%)	366 (36.5%)	
Often	1,804 (9.6%)	5,730 (10.2%)	1,843 (10.2%)	92 (9.2%)	
Daytime sound level of noise pollution, mean (SD), dB	55.3 ± 4.2	55.3 ± 4.2	55.3 ± 4.2	55.2 ± 4.0	.043
Evening sound level of noise pollution, mean (SD), dB	51.6 ± 4.2	51.6 ± 4.2	51.6 ± 4.2	51.4 ± 4.0	.043

Night sound level of noise pollution, mean (SD), dB	46.5 ± 4.2	46.5 ± 4.2	46.5 ± 4.2	46.3 ± 4.0	.043
16-hour sound level of noise pollution, mean (SD), dB	54.4 ± 4.2	54.4 ± 4.2	54.4 ± 4.2	54.2 ± 4.0	.043
24-hour sound level of noise pollution, mean (SD), dB	56.0 ± 4.2	56.0 ± 4.2	56.0 ± 4.2	55.8 ± 4.0	.043
Lifestyle factor					
Loud music exposure frequency, No. (%)					.303
No	21,320 (88.0%)	64,620 (88.0%)	20,480 (87.9%)	1,081 (88.0%)	
Yes, for less than a year	736 (3.0%)	2,225 (3.0%)	731 (3.1%)	36 (2.9%)	
Yes, for around 1-5 years	1,159 (4.8%)	3,341 (4.5%)	1,127 (4.8%)	65 (5.3%)	
Yes, for more than 5 years	1,018 (4.2%)	3,247 (4.4%)	974 (4.2%)	46 (3.7%)	
Time spent watching television, mean (SD), h	2.8 ± 1.6	2.8 ± 1.6	2.8 ± 1.6	2.8 ± 1.7	
Plays computer games, No. (%)					<.001
Rarely/never	59,065 (78.5%)	175,563 (77.8%)	55,173 (77.2%)	2,941 (78.3%)	
Sometimes	13,564 (18.0%)	41,700 (18.5%)	13,475 (18.9%)	686 (18.3%)	
Often	2,659 (3.5%)	8,497 (3.8%)	2,821 (3.9%)	131 (3.5%)	
Sleeplessness/insomnia, No. (%)					.702
Rarely/never	16,568 (22.0%)	49,346 (21.9%)	15,573 (21.8%)	849 (22.6%)	
Sometimes	35,738 (47.5%)	106,874 (47.4%)	33,878 (47.4%)	1,760 (46.8%)	
Usually	22,954 (30.5%)	69,485 (30.8%)	21,996 (30.8%)	1,153 (30.6%)	
Alcohol drinker status, No. (%)					.062
Never	2,211 (2.9%)	6,883 (3.1%)	2,227 (3.1%)	131 (3.5%)	
Previous	2,535 (3.4%)	7,538 (3.3%)	2,506 (3.5%)	118 (3.1%)	
Current	70,493 (93.7%)	211,231 (93.6%)	66,711 (93.4%)	3,512 (93.4%)	
Smoking status, No. (%)					.100
Never	40,882 (54.5%)	122,623 (54.5%)	38,652 (54.3%)	2,043 (54.5%)	
Previous	26,409 (35.2%)	79,824 (35.5%)	25,484 (35.8%)	1,316 (35.1%)	
Current	7,773 (10.4%)	22,682 (10.1%)	7,109 (10.0%)	393 (10.5%)	
Laboratory result					
Total cholesterol, mean (SD), mmol/l	221.0 ± 44.3	220.6 ± 44.1	220.6 ± 44.5	221.9 ± 44.8	.091
Triglycerides, mean (SD), mmol/l	154.5 ± 90.4	155.5 ± 90.4	156.9 ± 91.5	160.2 ± 91.6	<.001
HDL cholesterol, mean (SD), mmol/l	56.3 ± 14.8	56.0 ± 14.8	55.9 ± 14.7	55.7 ± 15.0	<.001
LDL cholesterol, mean (SD), mmol/l	138.0 ± 33.7	137.8 ± 33.6	137.9 ± 33.8	138.9 ± 33.7	.210
Major chronic comorbidity					
Hypercholesterolaemia, No. (%)	13,761 (18.3%)	41,503 (18.4%)	13,520 (18.9%)	710 (18.9%)	.005
Hypertension, No. (%)	22,484 (29.9%)	67,137 (29.7%)	21,150 (29.6%)	1,117 (29.7%)	.734
Heart failure, No. (%)	514 (0.7%)	1,582 (0.7%)	499 (0.7%)	24 (0.6%)	.929
Chronic kidney disease, No. (%)	1,131 (1.5%)	3,453 (1.5%)	1,101 (1.5%)	53 (1.4%)	.863
Any stroke, No. (%)	1,602 (2.1%)	4,732 (2.1%)	1,550 (2.2%)	64 (1.7%)	.207
Diabetic hypoglycemia, No. (%)	351 (0.5%)	1,343 (0.6%)	461 (0.6%)	16 (0.4%)	<.001
Type 2 diabetes mellitus, No. (%)	2,977 (4.2%)	9,016 (4.2%)	2,851 (4.2%)	127 (3.6%)	.257
Coronary artery disease, No. (%)	4,137 (5.5%)	13,045 (5.8%)	4,183 (5.9%)	211 (5.6%)	.013
Medication					
Use of ototoxic drugs, No. (%) (aspirin and ibuprofen consumption)	11,864 (22.2%)	35,804 (22.4%)	11,546 (22.9%)	592 (22.7%)	.036

Abbreviations: ARHL, age-related hearing loss; SD, standard deviation.

Table S2. Demographic comparison of the 85,588 participants included in the composite HLS analysis versus the remaining population within the UK Biobank.

	**Participants in the HLS analysis** (n=85,588)	**Participants not included in the HLS analysis** (n=290,876)	***P*-value**
Age, mean (SD), y	57.9 ± 7.9	57.4 ± 8.0	<.001
Sex, No. (%)			<.001
Male	42,865 (50.1%)	131,340 (45.2%)	
Female	42,723 (49.9%)	159,536 (54.8%)	
Education years, mean (SD), y	14.3 ± 5.0	13.7 ± 5.1	<.001
Number in household, mean (SD)	2.4 ± 1.2	2.4 ± 1.3	.639
Townsend deprivation index, mean (SD)	-1.6 ± 2.7	-1.6 ± 3.0	.727
Average total household income before tax			<.001
Less than £18,000	14,262 (19.0%)	57,335 (23.0%)	
1,8000 to 30,999£	19,300 (25.7%)	64,398 (25.9%)	
31,000 to 51,999£	20,308 (27.1%)	65,246 (26.2%)	
52,000 to 100,000£	16,470 (21.9%)	49,759 (20.0%)	
Greater than 100,000£	4,697 (6.3%)	12,297 (4.9%)	

Abbreviations: SD, standard deviation; HLS, healthy lifestyle score.

Table S3. Characteristics of participants in the Penn Medicine Biobank.

	Total (n=36,357)	Control (n=33,961)	ARHL case (n=2,396)	*P*-value*
**Age, mean (SD), y**	55.7 ± 16.4	55.0 ± 16.4	66.0 ± 11.3	<.001
**Sex, No. (%)**				<.001
**Male**	18,163 (50.0%)	16,841 (49.6%)	1,322 (55.2%)	
**Female**	18,194 (50.0%)	17,120 (50.4%)	1,074 (44.8%)	
**Ancestry, No. (%)**				<.001
**European**	26,523 (73.0%)	24,692 (72.7%)	1,831 (76.4%)	
**African American**	9,834 (27.0%)	9,269 (27.3%)	565 (23.6%)	

**P*-value indicates the significance of the difference between the control and ARHL case groups.
Abbreviations: ARHL, age-related hearing loss; SD, standard deviation.

Table S4. Odds ratio for ARHL associated with genetic risk group in the UK Biobank and Penn Medicine Biobank.

	UK Biobank		Penn Medicine Biobank					
**Population**	**European**		**Total**		**European**		**African American**	
**No. ARHL cases/ Total No.**	87,066/376,464		2,396/36,357		1,831/26,523		565/9,834	
**Genetic risk**	**OR (95% CI)**	***P*-value**	**OR (95% CI)**	***P*-value**	**OR (95% CI)**	***P*-value**	**OR (95% CI)**	***P*-value**
**Low**	1 (reference)		1 (reference)		1 (reference)		1 (reference)	
**Intermediate**	1.16 (1.14-1.19)	<.001	1.12 (1.00-1.25)	.054	1.14 (1.00-1.30)	.056	0.91 (0.73-1.15)	.444
**High**	1.36 (1.33-1.40)	<.001	1.37 (1.18-1.58)	<.001	1.38 (1.18-1.61)	<.001	1.22 (0.93-1.61)	.147
**Very high**	1.58 (1.47-1.70)	<.001	2.22 (1.55-3.18)	.001	1.89 (1.24-2.88)	.003	1.49 (0.73-3.05)	.278
**perSD increase**	1.12 (1.11-1.13)	<.001	1.14 (1.09-1.20)	<.001	1.14 (1.08-1.19)	<.001	1.10 (1.01-1.20)	.032

All analyses were adjusted by age, sex, genotype array, and PC 1 to 10.

Abbreviations: ARHL, age-related hearing loss; SD, standard deviation; OR, Odds ratio; CI, confidence interval; PC, principal component.

Table S5. Proportion of the variance explained in ARHL by different PRS methods.

Method	Parameter		UK Biobank	PMBB (EUR)	PMBB (AA)
PRS-CS	auto		**0.330**	**0.257**	**0.126**
LDpred2	auto		0.196	0.140	0.092
lassosum	shrinkage parameter: 0.2, 0.5, 0.9 and 1;	penalty parameter (λ): varied from 0.001 to 0.1	0.120	0.030	0.026
PRSice-2	**Pruning (r^2)**	**Threshold (*P*-value)**			
	0.1	5.00E-08	0.057	0.073	0.082
		1.00E-06	0.085	0.078	0.037
		1.00E-05	0.092	0.157	0.031
		1.00E-04	**0.117**	**0.177**	**0.128**
		1.00E-02	0.091	0.055	0.000
		5.00E-02	0.035	0.094	0.025
	0.5	5.00E-08	0.024	0.029	0.102
		1.00E-06	0.033	0.041	0.054
		1.00E-05	0.038	0.107	0.071
		1.00E-04	**0.147**	**0.142**	**0.162**
		1.00E-02	0.068	0.040	0.000
		5.00E-02	0.038	0.086	0.031
	0.9	5.00E-08	0.020	0.029	0.102
		1.00E-06	0.029	0.041	0.054
		1.00E-05	0.035	0.107	0.071
		1.00E-04	**0.149**	**0.142**	**0.162**
		1.00E-02	0.054	0.040	0.000
		5.00E-02	0.035	0.086	0.028

The proportion of variance explained for PRS was computed as Nagelkerke's pseudo-R2. Abbreviations: ARHL, age-related hearing loss; PRS, polygenic risk score; PMBB, Penn Medicine Biobank; EUR, European; AA, African American.

Table S6. Cox proportional hazard model with age at ARHL onset in the Penn Medicine Biobank.

	Penn Medicine Biobank			
**HL genetic risk**	**Total no. of participants**	**No. of ARHL cases (%)**	**Adjusted HR (95% CI)**	***P*-value**
**Low**	7,280	446 (6.1%)	1 (reference)	
**Intermediate**	21,817	1,419 (6.5%)	1.10 (0.99-1.22)	.090
**High**	6,897	492 (7.1%)	1.31 (1.14-1.50)	<.001
**Very high**	363	39 (10.7%)	1.93 (1.38-2.70)	<.001
**perSD increase**			1.13 (1.08-1.18)	<.001

HL genetic risk groups: low (<20th percentile), intermediate (20–80th percentile), high (80–99th percentile), and very high (>99th percentile). Cox proportional hazard model was adjusted by age, sex, genotype array, ancestry, and PC 1 to 10.

Abbreviations: ARHL, age-related hearing loss; HL, hearing loss; HR, Hazard ratio; CI, confidence interval; PC, principal component.

Table S7. Incidence rates of ARHL according to HL PRS risk and age groups in the Penn Medicine Biobank.

ARHL onset age	HL PRS group	Case, No.	Total, No.	Absolute risk, %	Incidence rate 1000 person-years (95% CI)
All (Onset age ≥ 40 years)	All	2,338	29,116	8.03%	3.50 (3.36-3.65)
	Low risk	433	5,958	7.27%	3.07 (2.79-3.37)
	Intermediate risk	1,387	17,515	7.92%	3.45 (3.27-3.63)
	High risk	480	5,365	8.95%	4.08 (3.73-4.47)
	Very high risk	38	278	13.67%	6.12 (4.33-8.39)
Onset age 40-55 years	All	356	7,931	4.49%	4.97 (4.46-5.51)
	Low risk	64	1,470	4.35%	4.77 (3.67-6.09)
	Intermediate risk	209	4,781	4.37%	4.82 (4.19-5.52)
	High risk	75	1,595	4.70%	5.33 (4.20-6.69)
	Very high risk	8	85	9.41%	9.42 (4.07-18.57)
Onset age 56-70 years	All	1,139	13,764	8.28%	10.20 (9.61-10.80)
	Low risk	198	2,839	6.97%	8.41 (7.28-9.67)
	Intermediate risk	676	8,228	8.22%	10.10 (9.36-10.89)
	High risk	248	2,568	9.66%	12.26 (10.78-13.89)
	Very high risk	17	129	13.18%	15.92 (9.27-25.49)
Onset age > 70 years	All	843	7,421	11.36%	15.40 (14.39-16.49)
	Low risk	171	1,649	10.37%	14.25 (12.19-16.55)
	Intermediate risk	502	4,506	11.14%	14.95 (13.67-16.31)
	High risk	157	1,202	13.06%	18.12 (15.40-21.19)
	Very high risk	13	64	20.31%	29.41 (15.66-50.30)

HL genetic risk groups: low (<20th percentile), intermediate (20–80th percentile), high (80–99th percentile), and very high (>99th percentile).

Abbreviations: ARHL, age-related hearing loss; HL, hearing loss; PRS, polygenic risk score; CI, confidence interval.

Table S8. Associations between lifestyle and environmental factors and ARHL.

	Crude model			adjusted Model*		
	OR	(95% CI)	*P*-value	OR	(95% CI)	*P*-value
Age, mean (SD), y	1.047	(1.046-1.048)	<.001	1.046	(1.045-1.047)	<.001
Sex						
Male	1 [Reference]			1 [Reference]		
Female	0.584	(0.575-0.593)	<.001	0.591	(0.581-0.600)	<.001
Education years, mean (SD), y	0.993	(0.992-0.995)	<.001	1.000	(0.999-1.002)	.975
Number in household, mean (SD)	0.919	(0.912-0.925)	<.001	1.016	(1.010-1.023)	<.001
Townsend deprivation index, mean (SD)	1.010	(1.008-1.013)	<.001	1.018	(1.015-1.021)	<.001
Average total household income before tax						
Less than £18,000	1 [Reference]			1 [Reference]		
1,8000 to 30,999£	0.888	(0.868-0.909)	<.001	0.932	(0.910-0.954)	<.001
31,000 to 51,999£	0.768	(0.750-0.786)	<.001	0.913	(0.891-0.935)	<.001
52,000 to 100,000£	0.675	(0.658-0.692)	<.001	0.870	(0.847-0.894)	<.001
Greater than 100,000£	0.584	(0.559-0.609)	<.001	0.762	(0.729-0.797)	<.001
Body composition						
Body mass index, mean (SD), kg/m^2	1.113	(1.105-1.121)	<.001	1.083	(1.075-1.091)	<.001
Height, mean (SD), cm	1.128	(1.119-1.136)	<.001	0.944	(0.933-0.955)	<.001
Weight, mean (SD), kg	1.167	(1.159-1.176)	<.001	1.067	(1.058-1.076)	<.001
Waist circumference, mean (SD), cm	1.251	(1.241-1.260)	<.001	1.101	(1.092-1.111)	<.001
Hearing condition						
Tinnitus						
No, never	1 [Reference]			1 [Reference]		
Yes, but not now, but have in the past	1.906	(1.830-1.986)	<.001	1.971	(1.890-2.055)	<.001
Yes, now some of the time	2.930	(2.808-3.056)	<.001	2.796	(2.678-2.919)	<.001
Yes, now a lot of the time	4.670	(4.344-5.020)	<.001	4.279	(3.975-4.606)	<.001
Yes, now most or all of the time	7.278	(6.937-7.636)	<.001	6.387	(6.082-6.707)	<.001
Tinnitus severity/nuisance						
Not at all	1 [Reference]			1 [Reference]		
Slightly	1.482	(1.410-1.558)	<.001	1.516	(1.440-1.596)	<.001
Moderately	2.595	(2.432-2.770)	<.001	2.737	(2.559-2.927)	<.001
Severely	3.366	(2.959-3.829)	<.001	3.755	(3.290-4.286)	<.001
Environmental factor						
Workplace very noisy						
Rarely/never	1 [Reference]			1 [Reference]		
Sometimes	1.141	(1.104-1.178)	<.001	1.137	(1.100-1.175)	<.001
Often	1.688	(1.610-1.770)	<.001	1.623	(1.546-1.705)	<.001
Noisy workplace						
No	1 [Reference]			1 [Reference]		
Yes, for less than a year	1.419	(1.341-1.502)	<.001	1.330	(1.254-1.409)	<.001
Yes, for around 1-5 years	1.864	(1.769-1.964)	<.001	1.739	(1.648-1.835)	<.001
Yes, for more than 5 years	3.036	(2.930-3.147)	<.001	2.567	(2.472-2.666)	<.001
Lifestyle factor						

Loud music exposure frequency							
No	1 [Reference]			1 [Reference]			
Yes, for less than a year	1.222	(1.136-1.316)	<.001	1.489	(1.380-1.607)	<.001
Yes, for around 1-5 years	1.546	(1.459-1.637)	<.001	1.966	(1.851-2.088)	<.001
Yes, for more than 5 years	1.844	(1.741-1.954)	<.001	2.088	(1.965-2.218)	<.001
Plays computer games							
Rarely/never	1 [Reference]			1 [Reference]			
Sometimes	1.023	(1.004-1.044)	.021	1.083	(1.061-1.105)	<.001
Often	1.269	(1.222-1.318)	<.001	1.322	(1.272-1.374)	<.001
Sleeplessness/insomnia							
Rarely/never	1 [Reference]			1 [Reference]			
Sometimes	1.150	(1.127-1.174)	<.001	1.198	(1.173-1.224)	<.001
Usually	1.502	(1.470-1.535)	<.001	1.590	(1.555-1.626)	<.001
Alcohol intake frequency (categorical)							
Alcohol drinker status (never vs. ever)	1.160	(1.108-1.214)	<.001	1.149	(1.097-1.204)	<.001
Smoking status (never vs. ever)	1.326	(1.306-1.347)	<.001	1.181	(1.163-1.200)	<.001
Biomarker							
Systolic blood pressure, mean (SD), mmHg	1.082	(1.074-1.090)	<.001	0.931	(0.924-0.939)	<.001
Diastolic blood pressure, mean (SD), mmHg	1.011	(1.003-1.019)	<.001	0.958	(0.951-0.966)	<.001
Total cholesterol, mean (SD), mmol/l	0.933	(0.925-0.940)	<.001	0.972	(0.964-0.980)	<.001
Triglycerides, mean (SD), mmol/l	1.107	(1.099-1.115)	<.001	1.041	(1.033-1.049)	<.001
HDL cholesterol, mean (SD), mmol/l	0.851	(0.844-0.859)	<.001	0.927	(0.918-0.936)	<.001
LDL cholesterol, mean (SD), mmol/l	0.959	(0.952-0.967)	<.001	0.982	(0.975-0.990)	<.001
Major chronic comorbidity							
Hypercholesterolaemia	1.191	(1.183-1.200)	<.001	1.051	(1.043-1.059)	<.001
Hypertension	1.153	(1.145-1.162)	<.001	1.040	(1.032-1.048)	<.001
Heart failure	1.038	(1.031-1.046)	<.001	1.010	(1.003-1.017)	<.001
Chronic kidney disease	1.035	(1.028-1.042)	<.001	1.007	(1.000-1.015)	.043
Any stroke	1.065	(1.058-1.072)	<.001	1.031	(1.024-1.038)	<.001
Diabetic hypoglycemia	1.025	(1.017-1.032)	<.001	1.012	(1.004-1.019)	.002
Type 2 diabetes mellitus	1.078	(1.070-1.085)	<.001	1.024	(1.016-1.032)	<.001
Coronary artery disease	1.148	(1.140-1.156)	<.001	1.062	(1.055-1.070)	<.001
Medications							
Use of ototoxic drug (aspirin and ibuprofen consumption)	1.062	(1.055-1.070)	<.001	1.116	(1.092-1.140)	<.001

*Model was adjusted by age, sex, genotype array, and PCs 1 to 10.

Abbreviations: ARHL, age-related hearing loss; OR, odds ratio; SD, standard deviation.

Table S9. Significance of each lifestyle/environmental factor in multivariate regression analysis considering mutual adjustments.

**Lifestyle/Environmental factor** (health lifestyle status)	**OR**	**(95% CI)**	***P*-value**
Smoking history (Never)	1.087	(1.051-1.124)	<.001
Alcohol history (Rarely/never)	1.183	(1.063-1.317)	.002
Body mass index (<30 kg/m^2)	1.029	(1.012-1.047)	<.001
Sleeplessness/Insomnia (Rarely/never)	1.127	(1.109-1.144)	<.001
Use of ototoxic drugs, aspirin and/or ibuprofen (No)	1.041	(1.001-1.083)	.049
Plays computer games (Rarely/never)	1.059	(1.025-1.095)	<.001
Loud music listening (No)	1.233	(1.206-1.260)	<.001
Noisy workplace exposure (No)	1.318	(1.298-1.338)	<.001
**Genetic factor**			
HL PRS	1.121	(1.103-1.140)	<.001

Multivariate regression analysis:
ARHL: HL PRS + lifestyle/environmental factors + covariates (age + sex + PC1-10 + Genotype array)
Abbreviations: ARHL, age-related hearing loss; HL, hearing loss; PRS, polygenic risk score; OR, odds ratio; CI, confidence interval; PC, principal component.

Table S10. Significance of the interaction terms between each lifestyle/environmental factor and genetic risk group for ARHL.

lifestyle/environmental factor	Field ID	*P*-value for interaction term*
Loud music listening	4836	.875
Noisy workplace exposure	4825	.478
Smoking history	20116	.215
Alcohol history	20117	.838
Body mass index	21001	.979
Use of ototoxic drugs (aspirin and/or ibuprofen)	6154	.368
Sleeplessness/Insomnia	1200	.585
Plays computer games	2237	.352

*The *P*-value was obtained through a multiplicative interaction analysis.

Table S11. Odds ratio for ARHL associated with healthy lifestyle score (Ideal lifestyle group as a reference).

			Model 1		Model 2		Model 3		Model 4	
**Lifestyle**	**Total no. of participants**	**No. of ARHL cases (%)**	**OR (95% CI)**	***P*-value**	**OR (95% CI)**	***P*-value**	**OR (95% CI)**	***P*-value**	**OR (95% CI)**	***P*-value**
**Ideal**	6,389	936 (14.7%)	1 (reference)		1 (reference)		1 (reference)		1 (reference)	
**Intermediate**	49,094	10,064 (20.5%)	1.44 (1.34-1.55)	<.001	1.49 (1.37-1.61)	<.001	1.51 (1.38-1.66)	<.001	1.41 (1.29-1.55)	<.001
**Poor**	26,609	7,652 (28.7%)	2.11 (1.95-2.27)	<.001	2.18 (2.01-2.36)	<.001	2.25 (2.05-2.47)	<.001	1.97 (1.79-2.17)	<.001
**Very poor**	3,496	1,303 (37.3%)	3.03 (2.75-3.35)	<.001	3.09 (2.77-3.44)	<.001	3.30 (2.92-3.74)	<.001	2.66 (2.33-3.03)	<.001

Detailed information on how to generate a healthy lifestyle score shown in **Methods S5** and **S7**.
Model 1 was adjusted by age, sex, genotype array, and PC 1 to 10.
Model 2: Model 1 + education years + household income + Townsend deprivation index + number in household.
Model 3: Model 2 + body mass index + height + weight + waist circumference + systolic blood pressure + diastolic blood pressure + Total cholesterol + HDL cholesterol + LDL cholesterol + Triglycerides + Creatinine + eGFR + Fasting blood glucose + HbA1c.
Model 4: Model 3 + tinnitus + major chronic comorbidities (hypercholesterolaemia, hypertension, heart failure, chronic kidney disease, any stroke, diabetic hypoglycemia, type 2 diabetes mellitus, and coronary artery disease)
Abbreviations: ARHL, age-related hearing loss; SD, standard deviation; OR, Odds ratio; CI, confidence interval; PC, principal component.

Table S12. Comparison between lifestyle scores and metabolic syndrome status (Ideal lifestyle group as a reference).

	Healthy lifestyle score			AHA lifestyle score			MetS health score		
**Lifestyle**	**Total no. of participants /No. of ARHL cases (%)**	**OR (95% CI)**	***P*-value**	**Total no. of participants /No. of ARHL cases (%)**	**OR (95% CI)**	***P*-value**	**Total no. of participants /No. of ARHL cases (%)**	**OR (95% CI)**	***P*-value**
Crude									
**Ideal**	6,389/ 936 (14.7%)	1 (reference)		54,812/ 12,483 (22.77%)	1 (reference)		26,992/ 5,560 (20.60%)	1 (reference)	
**Intermediate**	49,094/ 10,064 (20.5%)	1.50 (1.40-1.62)	<.001	23,543/ 5,619 (23.87%)	1.06 (1.03-1.10)	.001	19,924/ 4,649 (23.33%)	1.17 (1.12-1.23)	<.001
**Poor**	26,609/ 7,652 (28.8%)	2.35 (2.18-2.53)	<.001	7,233/ 1,853 (25.62%)	1.17 (1.10-1.24)	<.001	26,752/ 6,922 (25.87%)	1.35 (1.29-1.40)	<.001
**Very poor**	3,496/ 1,303 (37.3%)	3.46 (3.14-3.82)	<.001	n/a	n/a	n/a	n/a	n/a	n/a
Adjusted Model									
**Ideal**	6,389/ 936 (14.7%)	1 (reference)		54,812/ 12,483 (22.77%)	1 (reference)		26,992/ 5,560 (20.60%)	1 (reference)	
**Intermediate**	49,094/ 10,064 (20.5%)	1.44 (1.34-1.55)	<.001	23,543/ 5,619 (23.87%)	1.06 (1.02-1.10)	.001	19,924/ 4,649 (23.33%)	0.98 (0.94-1.03)	.391
**Poor**	26,609/ 7,652 (28.7%)	2.11 (1.95-2.27)	<.001	7,233/ 1,853 (25.62%)	1.18 (1.11-1.25)	<.001	26,752/ 6,922 (25.87%)	1.06 (1.01-1.10)	.012
**Very poor**	3,496/ 1,303 (37.3%)	3.03 (2.75-3.35)	<.001	n/a	n/a	n/a	n/a	n/a	n/a

The adjusted Model was adjusted by age, sex, genotype array, and PC 1 to 10.

Abbreviations: ARHL, age-related hearing loss; SD, standard deviation; OR, Odds ratio; CI, confidence interval; PC, principal component.

Table S13. Odds ratio for ARHL according to genetic risk and sex.

			Model 1		Model 2		Model 3		Model 4	
**Genetic risk**	**Total no. of participants**	**No. of ARHL cases (%)**	**OR (95% CI)**	***P*-value**	**OR (95% CI)**	***P*-value**	**OR (95% CI)**	***P*-value**	**OR (95% CI)**	***P*-value**
Male										
**Low**	34,845	8,912 (25.6%)	1 (reference)		1 (reference)		1 (reference)		1 (reference)	
**Intermediate**	104,520	29,455 (28.2%)	1.14 (1.11-1.17)	<.001	1.13 (1.11-1.17)	<.001	1.13 (1.11-1.17)	<.001	1.17 (1.11-1.16)	<.001
**High**	33,098	10,310 (31.2%)	1.32 (1.28-1.37)	<.001	1.33 (1.28-1.38)	<.001	1.32 (1.27-1.38)	<.001	1.33 (1.27-1.38)	<.001
**Very high**	1,742	554 (31.8%)	1.38 (1.24-1.54)	<.001	1.35 (1.21-1.51)	<.001	1.30 (1.15-1.47)	<.001	1.30 (1.15-1.47)	<.001
**perSD increase**			1.11 (1.10-1.12)	<.001	1.11 (1.10-1.12)	<.001	1.11 (1.10-1.12)	<.001	1.11 (1.10-1.12)	<.001
Female										
**Low**	40,460	6,540 (16.2%)	1 (reference)		1 (reference)		1 (reference)		1 (reference)	
**Intermediate**	121,359	22,612 (18.6%)	1.19 (1.15-1.23)	<.001	1.17 (1.13-1.21)	<.001	1.16 (1.12-1.21)	<.001	1.16 (1.12-1.20)	<.001
**High**	38,418	8,162 (21.3%)	1.41 (1.36-1.46)	<.001	1.38 (1.33-1.44)	<.001	1.37 (1.31-1.43)	<.001	1.37 (1.31-1.43)	<.001
**Very high**	2,022	521 (25.8%)	1.82 (1.64-2.02)	<.001	1.81 (1.61-2.04)	<.001	1.75 (1.54-1.99)	<.001	1.75 (1.54-1.99)	<.001
**perSD increase**			1.14 (1.13-1.15)	<.001	1.13 (1.12-1.15)	<.001	1.13 (1.11-1.15)	<.001	1.13 (1.11-1.15)	<.001

Model 1 was adjusted by age, genotype array, and PCs 1 to 10.

Model 2: Model 1 + education years + household income + Townsend deprivation index + number in household.

Model 3: Model 2 + body mass index + height + weight + waist circumference + systolic blood pressure + diastolic blood pressure + Total cholesterol + HDL cholesterol + LDL cholesterol + Triglycerides + Creatinine + eGFR + Fasting blood glucose + HbA1c.

Model 4: Model 3 + tinnitus + major chronic comorbidities (hypercholesterolaemia, hypertension, heart failure, chronic kidney disease, any stroke, diabetic hypoglycemia, type 2 diabetes mellitus, and coronary artery disease)

Abbreviations: ARHL, age-related hearing loss; SD, standard deviation; OR, Odds ratio; CI, confidence interval; PC, principal component.

Table S14. Odds ratio for ARHL according to genetic risk and tinnitus history.

			Model 1		Model 2		Model 3		Model 4	
**Genetic risk**	**Total no. of participants**	**No. of ARHL cases (%)**	**OR (95% CI)**	***P*-value**	**OR (95% CI)**	***P*-value**	**OR (95% CI)**	***P*-value**	**OR (95% CI)**	***P*-value**
History of Tinnitus										
**Low**	6,669	2,494 (37.4%)	1 (reference)		1 (reference)		1 (reference)		1 (reference)	
**Intermediate**	21,337	8,766 (41.1%)	1.17 (1.11-1.24)	<.001	1.18 (1.11-1.26)	<.001	1.20 (1.12-1.28)	<.001	1.19 (1.11-1.28)	<.001
**High**	7,097	3,115 (43.9%)	1.31 (1.23-1.41)	<.001	1.34 (1.24-1.44)	<.001	1.34 (1.23-1.46)	<.001	1.33 (1.22-1.45)	<.001
**Very high**	378	185 (48.9%)	1.59 (1.29-1.97)	<.001	1.63 (1.30-2.05)	<.001	1.78 (1.38-2.31)	<.001	1.79 (1.38-2.31)	<.001
**perSD increase**			1.11 (1.09-1.14)	<.001	1.12 (1.09-1.14)	<.001	1.12 (1.09-1.15)	<.001	1.12 (1.09-1.15)	<.001
No history of Tinnitus										
**Low**	17,410	2,771 (15.9%)	1 (reference)		1 (reference)		1 (reference)		1 (reference)	
**Intermediate**	51,755	9,012 (17.4%)	1.12 (1.07-1.18)	<.001	1.11 (1.05-1.17)	<.001	1.09 (1.03-1.16)	<.001	1.09 (1.03-1.15)	<.001
**High**	16,175	3,230 (20.0%)	1.34 (1.26-1.41)	<.001	1.35 (1.27-1.43)	<.001	1.34 (1.25-1.43)	<.001	1.34 (1.25-1.43)	<.001
**Very high**	841	187 (22.2%)	1.54 (1.30-1.83)	<.001	1.55 (1.29-1.86)	<.001	1.42 (1.15-1.75)	<.001	1.41 (1.15-1.74)	<.001
**perSD increase**			1.12 (1.10-1.14)	<.001	1.12 (1.09-1.14)	<.001	1.11 (1.09-1.13)	<.001	1.11 (1.09-1.14)	<.001

Model 1 was adjusted by age, sex, genotype array, and PCs 1 to 10.

Model 2: Model 1 + education years + household income + Townsend deprivation index + number in household.

Model 3: Model 2 + body mass index + height + weight + waist circumference + systolic blood pressure + diastolic blood pressure + Total cholesterol + HDL cholesterol + LDL cholesterol + Triglycerides + Creatinine + eGFR + Fasting blood glucose + HbA1c.Model 4: Model 3 + major chronic comorbidities (hypercholesterolaemia, hypertension, heart failure, chronic kidney disease, any stroke, diabetic hypoglycemia, type 2 diabetes mellitus, and coronary artery disease)

Abbreviations: ARHL, age-related hearing loss; SD, standard deviation; OR, Odds ratio; CI, confidence interval; PC, principal component.

Table S15. Odds ratio for ARHL according to Healthy lifestyle score and sex.

			Model 1		Model 2		Model 3		Model 4	
**Genetic risk**	**Total no. of participants**	**No. of ARHL cases (%)**	**OR (95% CI)**	***P*-value**	**OR (95% CI)**	***P*-value**	**OR (95% CI)**	***P*-value**	**OR (95% CI)**	***P*-value**
Male										
**Very poor**	2,673	1,065 (39.8%)	1 (reference)		1 (reference)		1 (reference)		1 (reference)	
**Poor**	15,904	5,249 (33.0%)	0.70 (0.64-0.76)	<.001	0.71 (0.64-0.77)	<.001	0.67 (0.60-0.74)	<.001	0.73 (0.65-0.81)	<.001
**Intermediate**	21,507	5,314 (24.7%)	0.47 (0.43-0.51)	<.001	0.48 (0.44-0.52)	<.001	0.44 (0.40-0.49)	<.001	0.52 (0.46-0.58)	<.001
**Ideal**	2,781	476 (17.1%)	0.31 (0.27-0.35)	<.001	0.32 (0.28-0.37)	<.001	0.30 (0.26-0.35)	<.001	0.38 (0.32-0.44)	<.001
Female										
**Very poor**	823	238 (28.9%)	1 (reference)		1 (reference)		1 (reference)		1 (reference)	
**Poor**	10,705	2,403 (22.5%)	0.68 (0.58-0.79)	<.001	0.70 (0.59-0.84)	<.001	0.69 (0.57-0.84)	<.001	0.76 (0.62-0.94)	.010
**Intermediate**	27,587	4,750 (17.2%)	0.48 (0.41-0.56)	<.001	0.49 (0.42-0.59)	<.001	0.48 (0.39-0.58)	<.001	0.56 (0.46-0.69)	<.001
**Ideal**	3,608	460 (12.8%)	0.35 (0.30-0.43)	<.001	0.33 (0.27-0.40)	<.001	0.30 (0.24-0.38)	<.001	0.38 (0.30-0.48)	<.001

Model 1 was adjusted by age, genotype array, and PCs 1 to 10.

Model 2: Model 1 + education years + household income + Townsend deprivation index + number in household.

Model 3: Model 2 + body mass index + height + weight + waist circumference + systolic blood pressure + diastolic blood pressure + Total cholesterol + HDL cholesterol + LDL cholesterol + Triglycerides + Creatinine + eGFR + Fasting blood glucose + HbA1c.

Model 4: Model 3 + tinnitus + major chronic comorbidities (hypercholesterolaemia, hypertension, heart failure, chronic kidney disease, any stroke, diabetic hypoglycemia, type 2 diabetes mellitus, and coronary artery disease)

Abbreviations: ARHL, age-related hearing loss; SD, standard deviation; OR, Odds ratio; CI, confidence interval; PC, principal component.

Table S16. Odds ratio for ARHL according to Healthy lifestyle score and tinnitus history.

			Model 1		Model 2		Model 3		Model 4	
**Genetic risk**	**Total no. of participants**	**No. of ARHL cases (%)**	**OR (95% CI)**	***P*-value**	**OR (95% CI)**	***P*-value**	**OR (95% CI)**	***P*-value**	**OR (95% CI)**	***P*-value**
History of Tinnitus										
**Very poor**	1,480	755 (51.0%)	1 (reference)		1 (reference)		1 (reference)		1 (reference)	
**Poor**	8,395	3,721 (44.3%)	0.77 (0.69-0.86)	<.001	0.79 (0.70-0.89)	<.001	0.80 (0.70-0.92)	0.002	0.80 (0.69-0.92)	0.002
**Intermediate**	11,508	4,216 (36.6%)	0.59 (0.52-0.66)	<.001	0.60 (0.53-0.68)	<.001	0.63 (0.55-0.73)	<.001	0.62 (0.54-0.72)	<.001
**Ideal**	1,068	335 (31.4%)	0.48 (0.40-0.57)	<.001	0.47 (0.39-0.57)	<.001	0.49 (0.40-0.60)	<.001	0.48 (0.39-0.60)	<.001
No history of Tinnitus										
**Very poor**	1,934	525 (27.2%)	1 (reference)		1 (reference)		1 (reference)		1 (reference)	
**Poor**	17,765	3,807 (21.4%)	0.75 (0.67-0.84)	<.001	0.76 (0.67-0.85)	<.001	0.72 (0.63-0.82)	<.001	0.71 (0.63-0.81)	<.001
**Intermediate**	37,019	5,727 (15.5%)	0.54 (0.48-0.60)	<.001	0.54 (0.48-0.60)	<.001	0.50 (0.44-0.57)	<.001	0.49 (0.43-0.56)	<.001
**Ideal**	5,259	593 (11.3%)	0.39 (0.34-0.44)	<.001	0.37 (0.32-0.43)	<.001	0.34 (0.29-0.41)	<.001	0.34 (0.28-0.40)	<.001

Model 1 was adjusted by age, sex, genotype array, and PCs 1 to 10.
Model 2: Model 1 + education years + household income + Townsend deprivation index + number in household.
Model 3: Model 2 + body mass index + height + weight + waist circumference + systolic blood pressure + diastolic blood pressure + Total cholesterol + HDL cholesterol + LDL cholesterol + Triglycerides + Creatinine + eGFR + Fasting blood glucose + HbA1c.
Model 4: Model 3 + major chronic comorbidities (hypercholesterolaemia, hypertension, heart failure, chronic kidney disease, any stroke, diabetic hypoglycemia, type 2 diabetes mellitus, and coronary artery disease)
Abbreviations: ARHL, age-related hearing loss; SD, standard deviation; OR, Odds ratio; CI, confidence interval; PC, principal component.

Figure S1. Study flowchart.

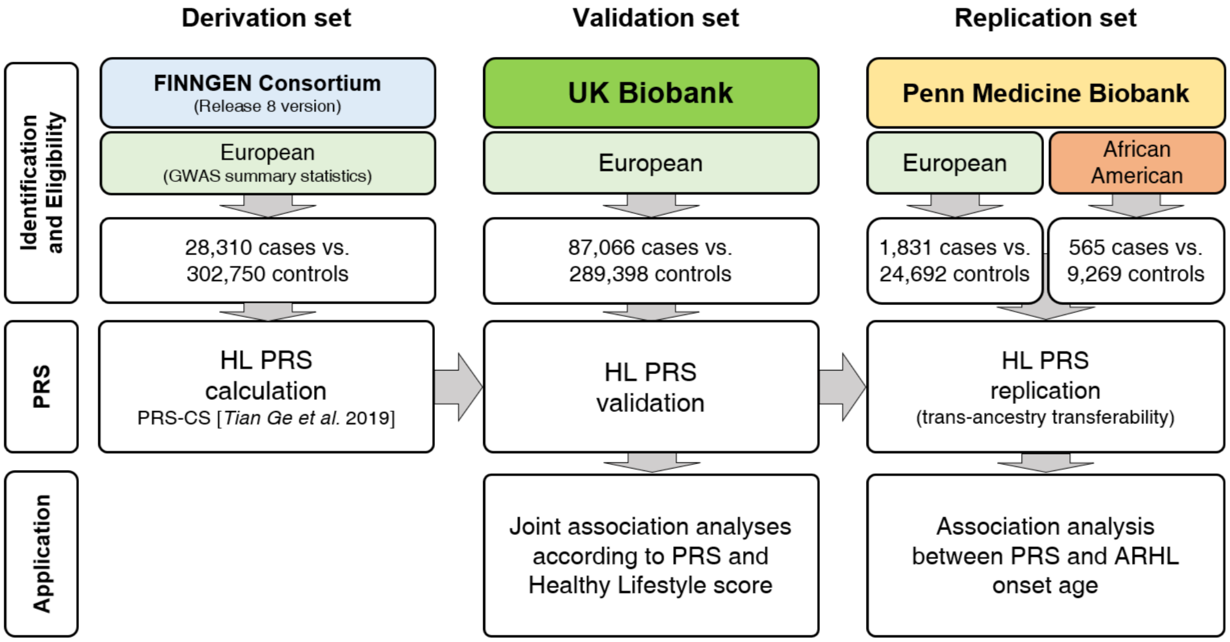

Abbreviations: ARHL, age-related hearing loss; PRS, Polygenic risk score.

Figure S2. Flowchart for generating a composite healthy lifestyle score in the UK Biobank.

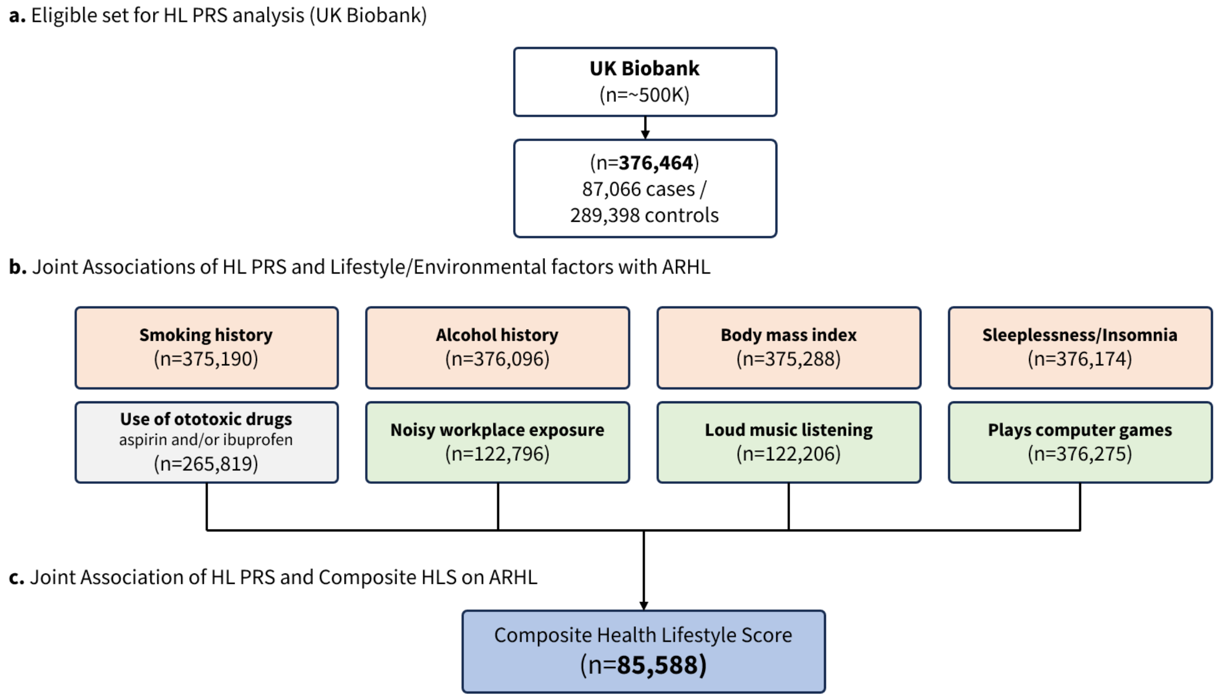

Abbreviations: HL, hearing loss; ARHL, age-related hearing loss; PRS, polygenic risk score; HLS, health lifestyle score.

Figure S3. Density and prevalence plots according to genetic risk for ARHL distribution in the UK Biobank.

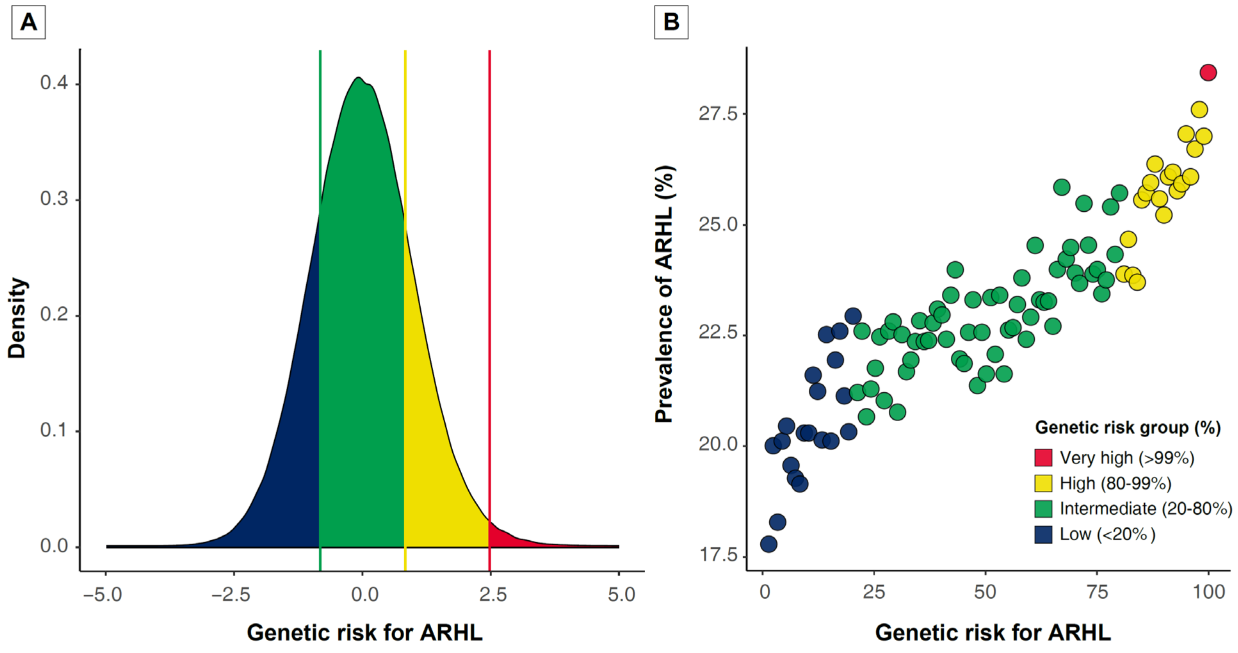

Abbreviation: ARHL, age-related hearing loss.

Figure S4. Cumulative incidence risk for onset age of ARHL in the Penn Medicine Biobank.

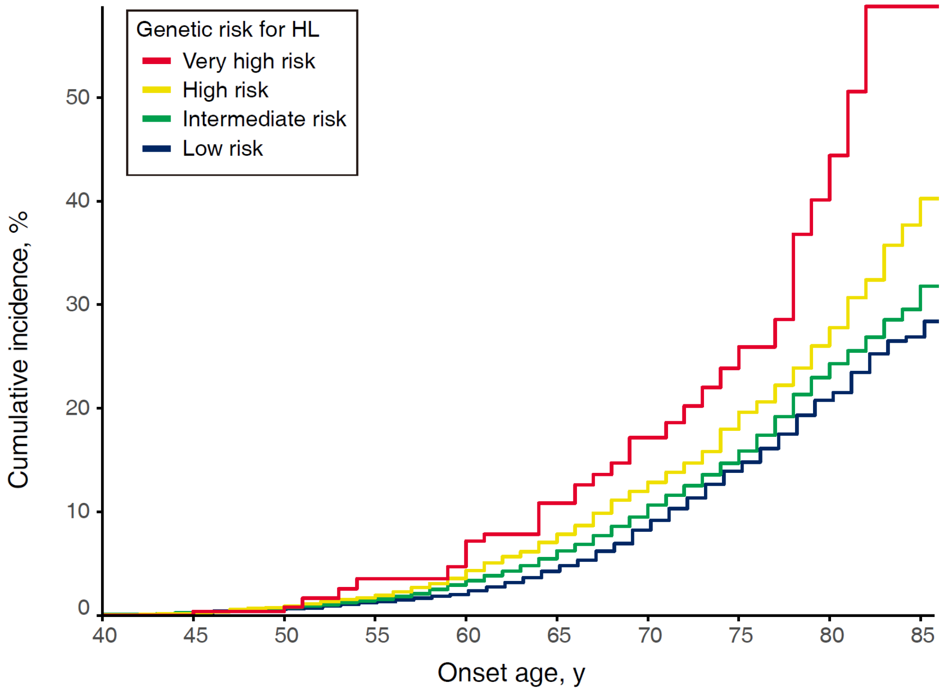

No. at genetic risk	40	45	50	55	60	65	70	75	80	85
Low risk	5925	5611	5134	4488	3642	2636	1649	892	424	146
Intermediate risk	17396	16332	14829	12734	10151	7312	4506	2496	1189	441
High risk	5329	4947	4436	3770	2906	2017	1202	650	292	98
Very high risk	277	262	238	193	154	111	64	37	14	2

Onset age, y

HL genetic risk groups: low (<20th percentile), intermediate (20–80th percentile), high (80–99th percentile), and very high (>99th percentile).
Abbreviations: HL, hearing loss; ARHL, age-related hearing loss.

Figure S5. Correlation matrix of lifestyle and environmental factors associated with ARHL.

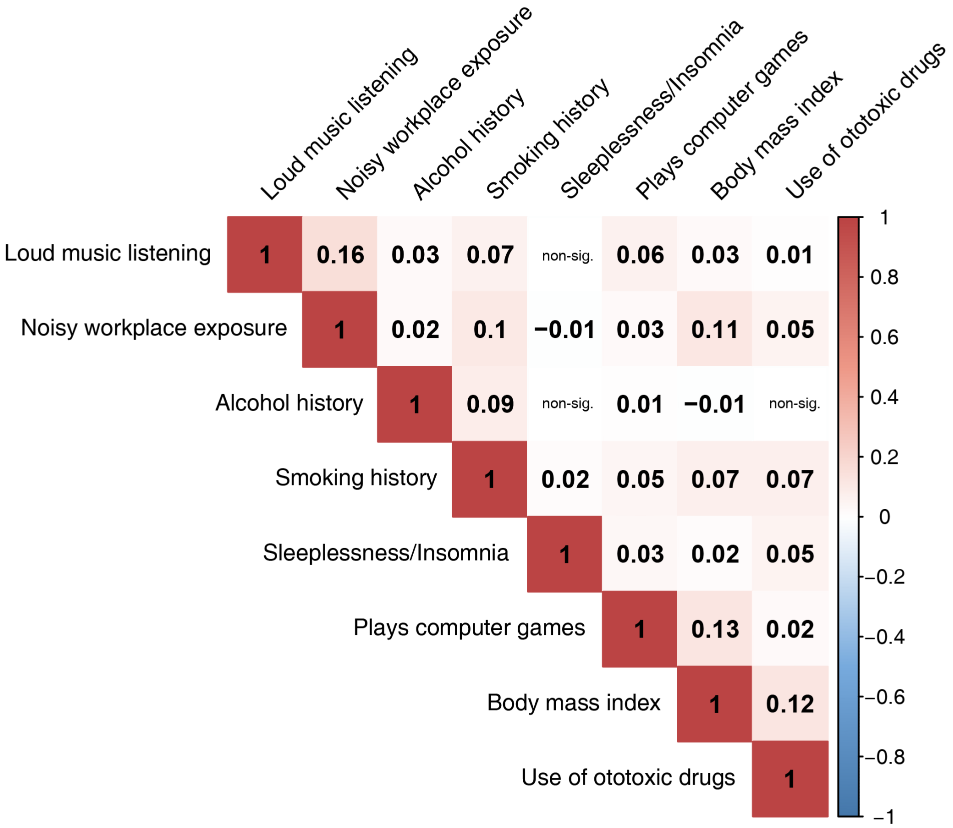

Supplement: Supplementary file 1 — Additional file 1: Methods S1-11, Tables S1-16, and Figures S1-5. Method S1. Penn Medicine Biobank banner author list and contribution statements. Method S2. Detailed definition of ARHL. Method S3. Detailed definitions of the covariates in the UK Biobank. Method S4. Detailed definitions of baseline major chronic comorbidities. Method S5. Detailed definitions of lifestyle factors, behaviors, and environmental factors in the UK Biobank. Method S6. Number of missing data for each variable in the UK Biobank. Method S7. Generating of composite healthy lifestyle score. Method S8. Detailed definitions of existing lifestyle score and metabolic syndrome status. Method S9. Detailed information on the genotype data quality control and imputation procedures. Method S10. Generating of polygenic risk score for ARHL. Method S11. Detailed information on statistical analysis. Table S1. Characteristics according to genetic risk group of ARHL in the UK Biobank; Table S2. Demographic comparison of the 85,588 participants included in the composite HLS analysis versus the remaining population within the UK Biobank. Table S3. Characteristics of participants in the Penn Medicine Biobank. Table S4. Odds ratio for ARHL associated with genetic risk group in the UK Biobank and Penn Medicine Biobank. Table S5. Proportion of the variance explained in ARHL by different PRS methods. Table S6. Cox proportional hazard model with age at ARHL onset in the Penn Medicine Biobank. Table S7. Incidence rates of ARHL according to HL PRS risk and age groups in the Penn Medicine Biobank. Table S8. Associations between lifestyle and environmental factors and ARHL. Table S9. Significance of each lifestyle/environmental factor in multivariate regression analysis considering mutual adjustments. Table S10. Significance of the interaction terms between each lifestyle/environmental factor and genetic risk group for ARHL. Table S11. Odds ratio for ARHL associated with healthy lifestyle score (Ideal lifestyle group as [file 12916_2024_3364_MOESM1_ESM.pdf]
